# A multi-field coupled model for gas migration in high-temperature coal seams considering fracture zones and fracture roughness

**DOI:** 10.1371/journal.pone.0349721

**Published:** 2026-09-08

**Authors:** Shantengyue Jiang, Mo Zhang

**Affiliations:** College of Mining, Liaoning Technical University, Fuxin, China; Henan Polytechnic University, CHINA

## Abstract

Gas migration in high-temperature coal seams is governed by fracture-zone connectivity, fracture-surface roughness, and thermo-mechanical deformation. However, fracture roughness is often treated qualitatively or absorbed into empirical permeability, which limits its use in field-scale gas-hazard assessment. This study develops a thermo-hydro-mechanical coupled model for gas-bearing coal seams with fracture zones. A dimensionless roughness parameter is introduced to quantify the relative asperity height of fracture surfaces and link fracture morphology with equivalent permeability, gas desorption, heat transfer, and coal-rock deformation. The model was validated using thermal-response data from the 16035 working face in the Liupanshui mining area and gas-production data from a coalbed methane well. It was then applied to analyze gas migration and coal-rock response in the 16035 working face. Results show that connected fracture zones act as preferential pathways for gas and heat transfer and control the spatial distributions of pressure, temperature, stress, and displacement near the roadway. Increasing the initial coal-seam temperature from 40°C to 50°C increases the maximum displacement by 15.8% and the maximum gas pressure by 4.3%. A lower roughness index also enhances gas transport. When the roughness index decreases from 0.4 to 0.1, the maximum permeability increases by 6.18%, and the gas desorption intensity at 3000s increases by 75.7%. These findings indicate that gas hazard in high-temperature coal seams should be evaluated by jointly considering fracture-zone geometry, quantifiable fracture roughness, and thermo-mechanical coupling effects.

## 1. Introduction

Gas control remains a critical issue in deep coal mining. This problem becomes more severe in high-temperature mines. Geothermal heat promotes gas release, heat accumulation, and deformation of the surrounding coal-rock mass [1–4]. Gas migration in deep coal seams is controlled by matrix diffusion, fracture seepage, and gas exchange between pores and fractures [5]. With increasing mining depth, abnormal gas emission and dynamic hazards become more likely [6–8]. Fracture zones provide preferential pathways for gas migration, accumulation, and release [9–11]. They also disturb the temperature field, deformation field, and stress field near roadways and working faces. Therefore, gas-hazard assessment in high-temperature mines must consider fracture-zone transport, thermal effects, and coal-rock response together [12–14].

Previous studies on high-temperature mines have mainly focused on thermal environment control, gas flow organization, and coal seam instability trend analysis [15–18]. Scalise et al. emphasized the thermal flywheel effect in climatic modelling for underground mines [19]. Yi et al. examined seasonal airflow effects on the thermal environment of deep mines [20]. Pan et al. studied pipeline layout and its influence on cooling performance [21]. Sunkpal assessed thermal comfort and cooling demand in deep underground mines [22]. These studies improved heat-control methods for deep mines. However, they mainly focused on airflow and cooling. The role of fracture zones in gas migration and hazard development was not fully examined [23–28].

Coupled gas flow, heat transfer, and deformation in fractured media have also received increasing attention. Temperature variation can change fracture permeability and alter gas-flow paths [15,16,29–31]. Thermo-mechanical loading can affect fracture-network evolution [32]. Coupled temperature-stress-gas models have improved gas-migration analysis in coal seams [33]. Experimental and numerical studies of fractured zones have also supported engineering design in high-temperature mines [34]. These studies confirm the strong interaction among seepage, heat transfer, and deformation. However, fracture morphology is still often simplified as a fixed aperture or permeability. The effect of fracture-surface roughness on gas seepage and gas desorption remains insufficiently quantified.

This limitation is important because gas migration in coal seams is controlled not only by the presence of fractures, but also by their connectivity, aperture, and surface roughness. Rough fracture surfaces reduce the effective hydraulic aperture, increase local flow resistance, and change the pressure difference between the coal matrix and fracture channels. Under high-temperature conditions, this process becomes more complex. Thermal expansion can narrow fracture openings, while higher temperature can promote methane desorption and gas migration. Therefore, fracture roughness may affect permeability evolution, gas desorption, heat transfer, and coal-rock stability at the same time.

To address these issues, this study develops a multi-field coupled model for gas migration in high-temperature coal seams with fracture zones. The model is based on established equations for deformation, gas seepage, heat transfer, and gas desorption. Its main contribution is the introduction of a quantifiable fracture roughness parameter into the coupled analysis. This parameter links fracture-surface morphology with equivalent permeability, gas-pressure redistribution, heat transfer, and methane desorption. The model was validated using field thermal-response data and gas-production data. It was then applied to the 16035 working face in the Liupanshui mining area. The analysis focuses on fracture-zone control, temperature effects, and roughness-dependent gas migration. The results provide a quantitative basis for gas-hazard assessment in high-temperature mines with dense fracture zones.

## 2. Study area and engineering background

The Liupanshui mining area, located in Guizhou Province, southwestern China, is a representative coal mining region characterized by relatively high underground temperatures, complex geological conditions, and increasing gas hazard risk. In recent years, gas emission in this mining area has shown an overall increasing trend with the expansion of mining extent and mining depth. Although no gas outburst events have been reported since the mine was established, gas release from broken coal, newly exposed coal-rock surfaces, pores, fractures, and goaf areas has gradually intensified during mining. This trend indicates that gas control has become increasingly important under deep and high-temperature mining conditions.

According to geological investigation reports and annual mine gas assessments, the absolute and relative methane emission rates have increased markedly since 2014. In particular, the mine changed from a low-gas mine in 2014–2015 to a high-gas mine from 2016 onward. Representative measurements in recent years further confirm the high-gas nature of the mine. For example, in 2022, the absolute methane emission rate was 34.00 m^3^/min and the relative methane emission rate was 4.40 m^3^/t, while the maximum absolute methane emission rates at the coal mining face and heading face were 21.50 and 3.00 m^3^/min, respectively. The long-term gas assessment results are summarized in Table 1. These data provide a direct engineering basis for investigating gas migration and hazard development in high-temperature coal mines.

**Table 1:** Annual mine gas classification results for the Liupanshui mining area.

Year	Mine methane emission, absolute (m³/min)	Mine methane emission, relative (m³/t)	Methane emission at coal face, absolute (m³/min)	Methane emission at heading face, absolute (m³/min)	Classification
2017	7.1	2.0	0.8	0.5	Low
2018	12.0	1.6	2.8	0.55	Low
2019	26.5	4.0	19.5	2.1	High
2020	35.0	4.5	24.0	1.4	High
2021	39.0	4.6	16.0	2.3	High
2022	39.5	4.8	29.5	3.0	High
2023	36.0	4.6	24.0	2.9	High
2024	40.0	5.0	20.8	2.3	High
2025	34.0	4.4	21.5	3.0	High

Coalfield geological exploration indicates that no major faults have been identified in the Liupanshui mining area. However, three-dimensional seismic exploration interpreted 18 faults within the mine field, with throws ranging from 4 to 20 m and generally falling within 6–10 m. Among them, three faults have throws greater than or equal to 12 m. Two-dimensional seismic exploration further showed that 12 of these faults are well constrained and reliable, accounting for 66.7% of the interpreted faults, while the remaining 6 are considered relatively reliable. Field observations during mining indicate that, although no major faults were exposed, numerous small faults are concentrated around roadways. This suggests that fracture zones are densely developed in underground workings and may form complex fracture networks. Such fracture networks can affect roadway stability, provide preferential pathways for gas migration, and intensify local hazard development under high-temperature and high-gas conditions.

Based on the above geological and gas-emission characteristics, the Liupanshui mining area provides a suitable engineering background for the present study. In particular, the 16035 working face contains multiple fracture zones and was therefore selected as the case study site for analyzing the coupled effects of fracture-zone development, fracture roughness, gas seepage, heat transfer, and coal-rock deformation. Under these conditions, conventional gas assessment that neglects fracture-zone behavior may underestimate gas accumulation risk and surrounding-rock instability. Therefore, it is necessary to quantitatively assess gas migration and coal-rock response by explicitly considering fracture-zone effects in this high-temperature mining environment.

## 3. Multi-field coupled model for a high-temperature coal seam with rough fracture zones

The model was established to describe gas migration in a high-temperature coal seam where fracture-zone distribution and fracture-surface morphology jointly affect transport and deformation. The coal-rock matrix was treated as a continuous porous medium, while fracture zones were represented as embedded conductive channels. Gas pressure, temperature, deformation, gas desorption, and fracture roughness were coupled through the permeability and flow terms.

The key difference from a conventional multi-field model is the treatment of fracture roughness. In many engineering-scale models, fractures are represented only by their position, length, aperture, or permeability. This simplification cannot distinguish a smooth fracture from a rough fracture when the nominal aperture is the same. In this study, the roughness effect is introduced through a dimensionless morphology parameter, *χ*, which reflects the relative height of surface asperities compared with the initial fracture aperture. By doing so, fracture roughness can be quantitatively transferred into the equivalent permeability and then into gas-pressure evolution, heat transfer, desorption intensity, and coal-rock deformation. This treatment provides a direct link between fracture morphology and gas-migration response under high-temperature mining conditions.

### 3.1. Governing equations for coal-rock deformation

The deformation of the coal-rock mass was described by the mechanical equilibrium equation for a porous medium. The constitutive equation included the effects of gas pressure, temperature, and adsorption. The volumetric strain was then expressed as a function of stress, gas pressure, and temperature. By combining these equations, the control equation for coal-rock deformation was obtained. This part of the model was used to calculate the stress and displacement fields during gas migration.

The differential stress equilibrium equation of porous medium coal and rock in mine roadway can be expressed as [16]:


$$\begin{array}{c} \sigma_{ij,j}+F_{i}=0 \end{array}$$ (1)

where $\sigma_{ij}$ is the total stress tensor of coal and rock, and $F_{i}$ is the volume force component. Considering factors such as gas pressure, temperature of coal and rock, adsorption effect, etc., the constitutive relationship of high temperature coal and rock mass can be expressed as [17]:


$$\begin{array}{c} \sigma_{ij}=2G\varepsilon_{ij}+\frac{2G\upsilon }{1-2\upsilon }\varepsilon_{kk}\delta_{ij}-\alpha p\delta_{ij}-K\alpha_{T}T\delta_{ij} \end{array}$$ (2)

where *G* is the shear modulus, $\varepsilon_{ij}$ is the strain of the coal-rock mass, *υ* is the Poisson’s ratio of the coal-rock mass at elevated temperature, *K* is the bulk modulus, *δ*_ij_ is the Kronecker delta, α is the Biot coefficient, *p* is the gas pressure within the coal-rock mass under high-temperature conditions, α_T_ is the thermal expansion coefficient, and *T* is the temperature of the coal-rock mass.

In addition, under the action of multiple factors, the volumetric strain of coal satisfies:


$$\begin{array}{c} \Delta \varepsilon_{V}=-\frac{\text{1}}{K}\left(\Delta \overset{―}{\sigma }-\alpha \Delta p\right)+\alpha_{T}\Delta T \end{array}$$ (3)

where $\varepsilon_{V}=\varepsilon_{11}+\varepsilon_{22}+\varepsilon_{33}$, $\overset{―}{\sigma }=-\sigma_{kk}/3$.

Combining the above equations yields the governing equation for the structural deformation of the coal-rock mass, which can be expressed as follows:


$$\begin{array}{c} Gu_{i,jj}+\frac{G}{1-2\upsilon }u_{j,ij}=\alpha p_{,i}+K\alpha_{T}T_{,i}-f_{i} \end{array}$$ (4)

### 3.2. Roughness-dependent permeability and gas flow

Gas flow in the coal matrix is governed by the evolution of fracture storage space, gas exchange between pores and fractures, and the hydraulic resistance of rough microfractures. In this study, the matrix is not treated as a smooth continuum with fixed permeability. Instead, the permeability of the matrix-scale fracture system is allowed to vary with gas pressure, coal-rock deformation, and fracture-surface roughness.

During gas migration, pressure redistribution changes the fracture pore volume and thus modifies the local porosity. The porosity evolution of the fracture system is described as [9,10]:


$$\begin{array}{c} d\phi =\frac{\text{1}}{K}(\alpha -\phi )(d\overset{―}{\sigma }+dp_{f}) \end{array}$$ (5)

where *p* is the gas pressure in the fracture. The corresponding normalized form is:


$$\begin{array}{c} \phi =\alpha -\left(\alpha -\phi_{\text{0}}\right)\cdot e^{\frac{\left(\overset{―}{\sigma }-\overset{―}{\sigma_{\text{0}}}\right)+\left(p-p_{\text{0}}\right)}{K}} \end{array}$$ (6)

where the subscript 0 represents the initial state before gas extraction. In Eqs. (5) and (6), ϕ is the fracture porosity, α is the Biot coefficient, K is the bulk modulus of the coal-rock skeleton, σ̄ is the mean stress, and p is the fracture gas pressure. Eq. (5) expresses the incremental change in fracture porosity caused by stress and gas-pressure variation. The factor $\left(\alpha -\phi_{\text{0}}\right)$ represents the compressible part of the fracture-pore system. The normalized form in Eq. (6) is obtained by integrating Eq. (5) from the initial state to the current state, with α and K treated as constants within each calculation step. The exponent in Eq. (6) is dimensionless. It represents the cumulative change in mean stress and gas pressure normalized by the bulk modulus. Therefore, Eq. (6) describes how stress redistribution and gas-pressure evolution jointly modify the available fracture storage space.

Methane in fractured coal occurs as free gas in fracture voids and adsorbed gas on the internal surfaces of pores and fractures. The total gas mass can therefore be expressed as [13–15]:


$$\begin{array}{c} m_{t}=\rho_{g}\phi +\rho_{ga}\rho_{s}\frac{V_{L}p_{m}}{p_{m}+p_{L}}+\rho_{ga}\rho_{c}v_{b} \end{array}$$ (7)

where $m_{t}$ is the total gas mass in the coal seam. The mass balance for gas transport in the fracture system is written as:


$$\begin{array}{c} Q_{g}=\frac{\partial m_{t}}{\partial t}+\nabla \cdot \left(\rho_{g}{𝐪}_{{\;}_{𝐠}}\right) \end{array}$$ (8)

where $Q_{g}$ is the gas source term and ${𝐪}_{{\;}_{𝐠}}$ is the seepage velocity in the fracture. For the pressure range considered here, the basic seepage velocity follows Darcy’s law:


$$\begin{array}{c} {𝐪}_{{\;}_{𝐠}}=-\frac{k}{\mu }\nabla p \end{array}$$ (9)

where *k* is the permeability of the coal-seam fracture system. Because gas flow through narrow and rough fractures is affected by additional hydraulic resistance, a Forchheimer correction is introduced into the flow relation [15,18]:


$$\begin{array}{c} -\nabla p_{f}=\frac{k}{\mu }\cdot {𝐪}_{{\;}_{𝐠}}+\rho_{g}\beta \cdot {𝐪}_{{\;}_{𝐠}}\cdot \left|{𝐪}_{{\;}_{𝐠}}\right|=\frac{k}{\mu }\cdot {𝐪}_{{\;}_{𝐠}}\cdot \left(1+\frac{\mu \cdot \rho_{g}\beta }{k}\cdot \left|{𝐪}_{{\;}_{𝐠}}\right|\right) \end{array}$$ (10)

The correction coefficient is given by [15,18]:


$$\begin{array}{c} \delta =\frac{\text{1}}{\left(1+\frac{\mu \cdot \rho_{g}\beta }{k}\cdot \left|{𝐪}_{{\;}_{𝐠}}\right|\right)} \end{array}$$ (11)

Accordingly, the gas flow rate in the coal seam can be obtained as:


$$\begin{array}{c} m_{t}=\rho_{g}{𝐪}_{{\;}_{𝐠}}=-\frac{k}{\mu }\nabla p\cdot \rho_{g}\cdot \delta \end{array}$$ (12)

Substitution of the storage term and the seepage term gives the governing equation for total gas flow:


$$\begin{array}{c} Q_{T}=\frac{\partial \left(\phi \cdot p\right)}{\partial t}\cdot \frac{\rho_{ga}}{p_{a}}+\frac{\partial }{\partial t}\left(\rho_{ga}\rho_{s}\cdot V_{g}\right)+\nabla \left(-\frac{k}{\mu }\nabla p\cdot \rho_{g}\cdot \delta \right)+\rho_{ga}\rho_{s}\frac{dv_{b}}{dt} \end{array}$$ (13)

In Eq. (13), $Q_{T}$ denotes the total gas-flow term in the coal seam. The first term represents the temporal change in free gas stored in the fracture-pore space. The second term represents the contribution of adsorbed gas in the coal matrix and its exchange with the fracture system. The divergence term describes gas convection through the fracture network, where the permeability *k* and the correction coefficient *δ* jointly control the Darcy-Forchheimer flow capacity. The last term represents the gas contribution associated with the change in adsorbed gas content. Therefore, Eq. (13) is a mass-balance equation that combines fracture gas storage, matrix-fracture gas exchange, pressure-driven seepage, and desorption-related gas supply.

The remaining key quantity in Eq. (13) is *k*. For rough microfractures, *k* cannot be represented only by the nominal fracture aperture, because asperities locally narrow the flow path and increase the contact obstruction between opposite fracture walls. To account for this effect, a rough-fracture permeability relation is introduced [9,15]:


$$\begin{array}{c} \left\{ \begin{array}{c} @\;l@\;k=k_{\text{0}}\cdot \frac{{\left[1-\frac{\sqrt{\text{2}}h}{a_{\text{0}}}ln\left(\frac{p}{p_{\text{0}}}\right)\right]}^{\text{3}}\times \left[1-b\left(p-p_{\text{0}}\right)\right]}{1+b\left(p-p_{\text{0}}\right)},b\neq 0\\ k=k_{\text{0}}\cdot {\left[1-\frac{\sqrt{\text{2}}h}{a_{\text{0}}}ln\left(\frac{p}{p_{\text{0}}}\right)\right]}^{\text{3}},b=0 \end{array}\right. \end{array}$$ (14)

where $a_{\text{0}}$ is the initial fracture aperture, *k*_*0*_ is the initial permeability of the fracture system, *h* is the root mean square height of fracture-surface asperities, *p*_*0*_ is the initial gas pressure in the fracture system, and *b* is the ratio of contact area to fracture area. This equation links fracture permeability with two different effects. The pressure ratio *p*/*p*_*0*_ describes the pressure-dependent change in the flow channel, whereas *h*/*a*_*0*_ describes the relative roughness of the fracture surface.

For the present coupled model, the roughness effect is introduced through the geometric ratio between asperity height and initial aperture. This treatment avoids using roughness as an independent fitting coefficient and keeps its physical meaning tied to fracture morphology [9,15,18]. The dimensionless fracture roughness parameter is therefore defined as:


$$\begin{array}{c} \chi =\sqrt{\text{2}}\cdot \frac{h}{a_{\text{0}}} \end{array}$$ (15)

**Fig 1:**
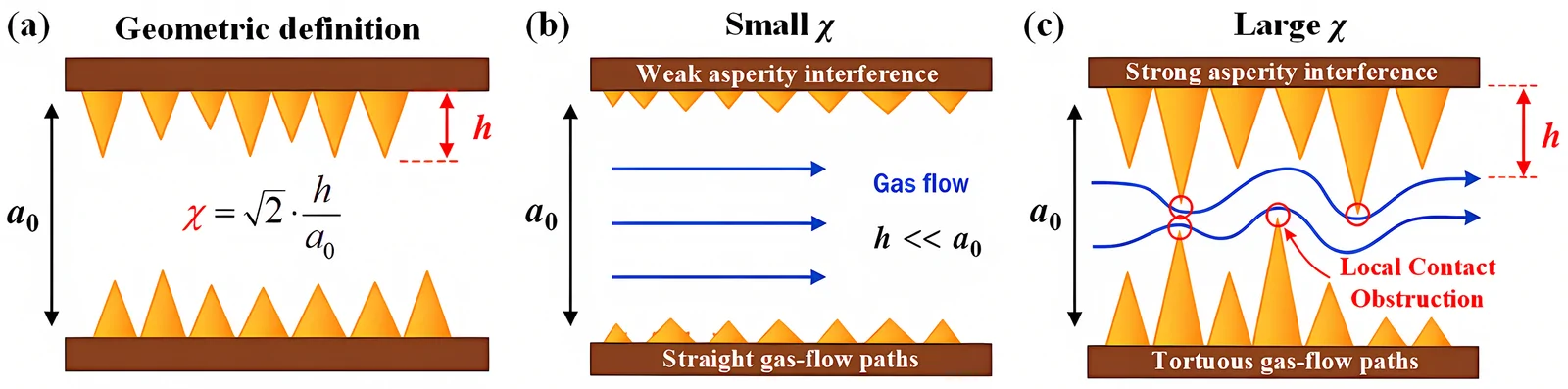
Physical meaning of the fracture roughness parameter.

Fig 1 illustrates the geometric meaning of the fracture roughness parameter defined in Eq. (15). In this definition, h denotes the root mean square height of the fracture-surface asperities, and a0 is the initial fracture aperture. Therefore, χ is a dimensionless parameter that describes the roughness amplitude normalized by the initial fracture aperture, rather than an independent empirical coefficient. For a given $a_{\text{0}}$, a larger χ indicates a rougher fracture surface, stronger asperity interference. This reduction in ae increases local flow obstruction and flow-path tortuosity, thereby weakening the gas-flow capacity of the fracture. Conversely, a smaller χ represents a smoother fracture surface with weaker asperity interference and a larger effective flow space.

The value of *χ* is not limited to a fixed universal range. It depends on the fracture-surface morphology and the initial fracture aperture. The values used in the following numerical cases are controlled roughness levels for comparing the effect of fracture roughness on permeability and gas desorption, rather than the complete range of *χ*. The physical interpretation of *χ* is monotonic: a larger χ indicates a rougher fracture surface and a stronger reduction in effective flow capacity.

The parameter *χ* is different from conventional roughness descriptors such as the joint roughness coefficient, fractal dimension, and self-affine fractal parameters. JRC describes the empirical roughness of a fracture profile. Fractal dimension and self-affine parameters describe the scale-dependent complexity and spatial correlation of fracture surfaces. These descriptors are useful for characterizing fracture morphology, but they do not directly show how much of the initial fracture aperture is occupied by asperity height. In contrast, *χ* directly normalizes the asperity height by the initial aperture. Therefore, it provides a more explicit and straightforward roughness measure for the present coupled seepage model. Through χ, the effect of fracture roughness can be directly introduced into the equivalent hydraulic aperture and permeability term, and can further affect gas-pressure redistribution, heat transfer, and methane desorption.

Substituting Eq. (15) into Eq. (14), the roughness effect then enters the gas-flow equation through the permeability-related flux term. By combining Eqs. (6), (13), and (14), the roughness-dependent gas-flow equation is obtained as follows:


$$\begin{array}{c} \left\{ \begin{array}{c} @\;l@\;Q_{T,f}=\left(\alpha -\phi_{\text{0}}\right)\cdot \frac{\partial \left(e^{\frac{\left(\overset{―}{\sigma }-\overset{―}{\sigma_{\text{0}}}\right)+\left(p-p_{\text{0}}\right)}{K}}\cdot p\right)}{\partial t}\cdot \frac{\rho_{ga}}{p_{a}}+\frac{\partial }{\partial t}\left(\rho_{ga}\rho_{s}\cdot V_{g}\right)+\\ \;\rho_{g}\cdot \nabla \left(-k_{\text{0}}\cdot \frac{{\left[1-\chi \cdot ln\left(\frac{p}{p_{\text{0}}}\right)\right]}^{\text{3}}\times \left[1-b\left(p-p_{\text{0}}\right)\right]}{1+b\left(p-p_{\text{0}}\right)\cdot \mu }\nabla p\cdot \delta \right)+\rho_{ga}\rho_{s}\frac{dv_{b}}{dt}\\ Q_{T,n}=\left(\alpha -\phi_{\text{0}}\right)\cdot \frac{\partial \left(e^{\frac{\left(\overset{―}{\sigma }-\overset{―}{\sigma_{\text{0}}}\right)+\left(p-p_{\text{0}}\right)}{K}}\cdot p\right)}{\partial t}\cdot \frac{\rho_{ga}}{p_{a}}+\frac{\partial }{\partial t}\left(\rho_{ga}\rho_{s}\cdot V_{g}\right)\\ \;+\rho_{g}\cdot \nabla \left(\frac{-k_{\text{0}}\cdot \nabla p\cdot \delta }{\mu }\times {\left[1-\chi \cdot ln\left(\frac{p}{p_{\text{0}}}\right)\right]}^{\text{3}}\right)+\rho_{ga}\rho_{s}\frac{dv_{b}}{dt} \end{array}\right. \end{array}$$ (16)

where $Q_{T,f}$ denotes the gas-flow equation with roughness-dependent permeability and contact-area correction when b ≠ 0, while $Q_{T,n}$ denotes the corresponding simplified form when b = 0. All other variables retain the definitions given above. The factor ${\left[1-\chi \cdot ln\left(\frac{p}{p_{\text{0}}}\right)\right]}^{\text{3}}$ is the key term linking fracture morphology with gas transport. It represents the reduction in equivalent hydraulic aperture caused by rough fracture surfaces. When χ =0, the roughness correction vanishes and the permeability term returns to the smooth-fracture reference form. Under the pressure conditions considered in this study, increasing χ strengthens the aperture-reduction effect and lowers the effective permeability of the fracture system.

### 3.3. Flow in fracture zones and stochastic fracture-network generation

Gas flow in the fracture zones was described separately from that in the matrix. For the fracture-zone domain, a seepage balance equation was introduced. The permeability of the macro-fractures followed the cubic law. In this way, the fracture zones were treated as discrete conductive channels in the coal-rock mass. This part of the model was used to describe the preferential flow of gas through the fracture network.

For the fracture zone, the seepage equilibrium equation is given by [18]:


$$\begin{array}{c} d_{f}\frac{\partial }{\partial t}\left(\rho_{w}\phi_{f2}\right)+\nabla_{T}\cdot \left(d_{f}\rho \frac{k_{f2}}{\mu }\nabla_{T}P\right)=d_{f}Q_{fm} \end{array}$$ (17)

where $d_{f}$ denotes the fracture width, $\nabla_{T}$ denotes the derivative along the fracture propagation direction, and $k_{f2}$ is the permeability of the macroscopic fracture:


$$\begin{array}{c} k_{f2}=\frac{{d_{f}}^{\text{2}}}{12} \end{array}$$ (18)

To represent the complex fracture distribution around the roadway, the fracture network was generated by a Monte Carlo method [19,20]. In this method, each fracture was defined by its center point, trace length, and orientation angle. The fracture orientation was generated from random variables through statistical transformation. Repeated sampling was then used to generate a stochastic fracture network for numerical simulation. This method is suitable for describing the small faults and fracture zones observed in the study area.

The coordinates of the fracture endpoints can be determined by three parameters, namely, the fracture center point, fracture trace length, and fracture orientation angle:


$$\begin{array}{c} \begin{array}{c} x=X_{\text{0}}\pm \left(\frac{l}{\text{2}}\right)cos\theta \\ y=Y_{\text{0}}\pm \left(\frac{l}{\text{2}}\right)sin\theta \end{array} \end{array}$$ (19)

If the fracture is treated as a line segment, the coordinates of its midpoint are $\left(X_{\text{0}},Y_{\text{0}}\right)$, l is the fracture length, and θ is the fracture orientation angle measured counter clockwise from the x-axis. The orientation of a single fracture can be generated by a uniform distribution transformation based on the Box-Muller principle:


$$\begin{array}{c} \gamma =\gamma_{\mu }+\gamma_{\sigma }\sqrt{-2lnF_{\text{1}}}cos\left(2\pi F_{\text{2}}\right) \end{array}$$ (20)

where $F_{\text{1}}$ and $F_{\text{2}}$ are random numbers uniformly distributed between 0 and 1. Using the above method, a set of random fractures can be generated.

### 3.4. Heat transfer equation and coupled solution

Because the coal seam is located in a high-temperature mining environment, heat transfer must be considered together with gas flow and coal-rock deformation. During gas migration, heat conduction occurs in the coal-rock mass, and heat is also transferred by the gas phase. The energy equation was therefore introduced to describe heat transfer during gas invasion. Under the assumption of local thermal equilibrium, the solid phase and gas phase were expressed by equivalent thermal parameters, and the final heat transfer equation was obtained.

For a high-temperature coal seam, during gas invasion, heat conduction can be described by the following equation [18]:


$$\begin{array}{c} \frac{\left(1-\varphi \right)\rho_{\text{s}}c_{\text{ps}}\partial T_{\text{a}}}{\partial t}+KT_{\text{s}}\alpha_{T}\frac{\partial \varepsilon_{V}}{\partial t}-\left(1-\varphi \right)\nabla \cdot \left({\overset{―}{\kappa }}_{\text{s}}\nabla T_{\text{s}}\right)=Q_{T\text{s}} \end{array}$$ (21)

$$\begin{array}{c} \frac{\phi \rho_{\text{g}}c_{\text{pg}}\partial T_{\text{g}}}{\partial t}+\rho_{\text{g}}c_{\text{pg}}\cdot v_{\text{g}}\nabla T_{\text{g}}-\phi \nabla \cdot \left({\overset{―}{\kappa }}_{\text{g}}\nabla T_{\text{g}}\right)=Q_{T\text{g}} \end{array}$$ (22)

where *s* and *g* denote the coal matrix and gas, respectively, *T*_*a*_ is the reference temperature, *c*_p_ is the specific heat capacity during gas invasion, $\overset{―}{\kappa }$ is the thermal conductivity, and *Q*_T_ is the heat generation during seepage.

In addition, according to the thermodynamic heat balance assumption, the above equations can be combined as:


$$\begin{array}{cc} & \frac{\partial }{\partial t}\left[{\left(\rho C_{p}\right)}_{c}T\right]=\nabla \cdot (\lambda \nabla T)-\alpha_{T}TK\cdot \frac{\partial \varepsilon_{S}}{\partial t}-q_{ad}\cdot \frac{\partial \varepsilon_{V}}{\partial t}\cdot \left(\frac{\rho_{s}\rho_{c}}{M_{c}}+\frac{\rho_{s}\rho_{\text{g}}}{M_{\text{g}}}\right)\\ & +\nabla T\cdot \left[k_{\text{0}}\cdot {\left[\text{1}-\chi \cdot \text{ln}\left(\frac{p}{p_{\text{0}}}\right)\right]}^{\text{3}}\times \frac{\mu_{c}+\mu_{\text{g}}}{\mu_{c}\cdot \mu_{\text{g}}}\cdot \left(\nabla p_{c}\rho_{c}C_{c}+\nabla p_{\text{g}}\rho_{\text{g}}C_{\text{g}}\right)\right] \end{array}$$ (23)

In Eq. (23), *(ρC*_*p*_*)*_*c*_ is the equivalent heat capacity of the coal seam, *ε*_*s*_ is the skeleton strain. The term containing *q*_*ad*_ represents the heat effect associated with methane adsorption and desorption, and *ε*_*v*_ is the volumetric strain. *M*_*c*_ and *M*_*g*_ are the molar masses of the coal matrix component and methane gas, respectively. *μ*_*c*_ and *μ*_*g*_ are the dynamic viscosities used in the coupled heat-advection term. The final term describes heat transport induced by gas flow through the rough-fracture permeability term. Thus, Eq. (23) couples heat conduction, solid deformation, adsorption/desorption heat, and seepage-induced heat transfer.

Therefore, the model for gas transport across the fracture zones in a high-temperature mine can be obtained from Eqs. (4), (6), (13), (15), (16), (17)-(20), and (23). The coupled model consists of the deformation equation of the coal-rock mass, the permeability equation considering fracture roughness, the gas-flow equation in the matrix, the seepage equation in the fracture zones, the stochastic fracture-network model, and the heat transfer equation. These equations were solved together to simulate gas migration, temperature variation, stress change, and deformation in a high-temperature coal seam containing fracture zones. The model was then used in the following sections to analyze gas migration, outburst tendency, and coal-rock stability in the study area.

## 4. Validation of the proposed rough-fracture multi-field coupled model

### 4.1. Validation based on thermal response at the 16035 working face

**Fig 2:**
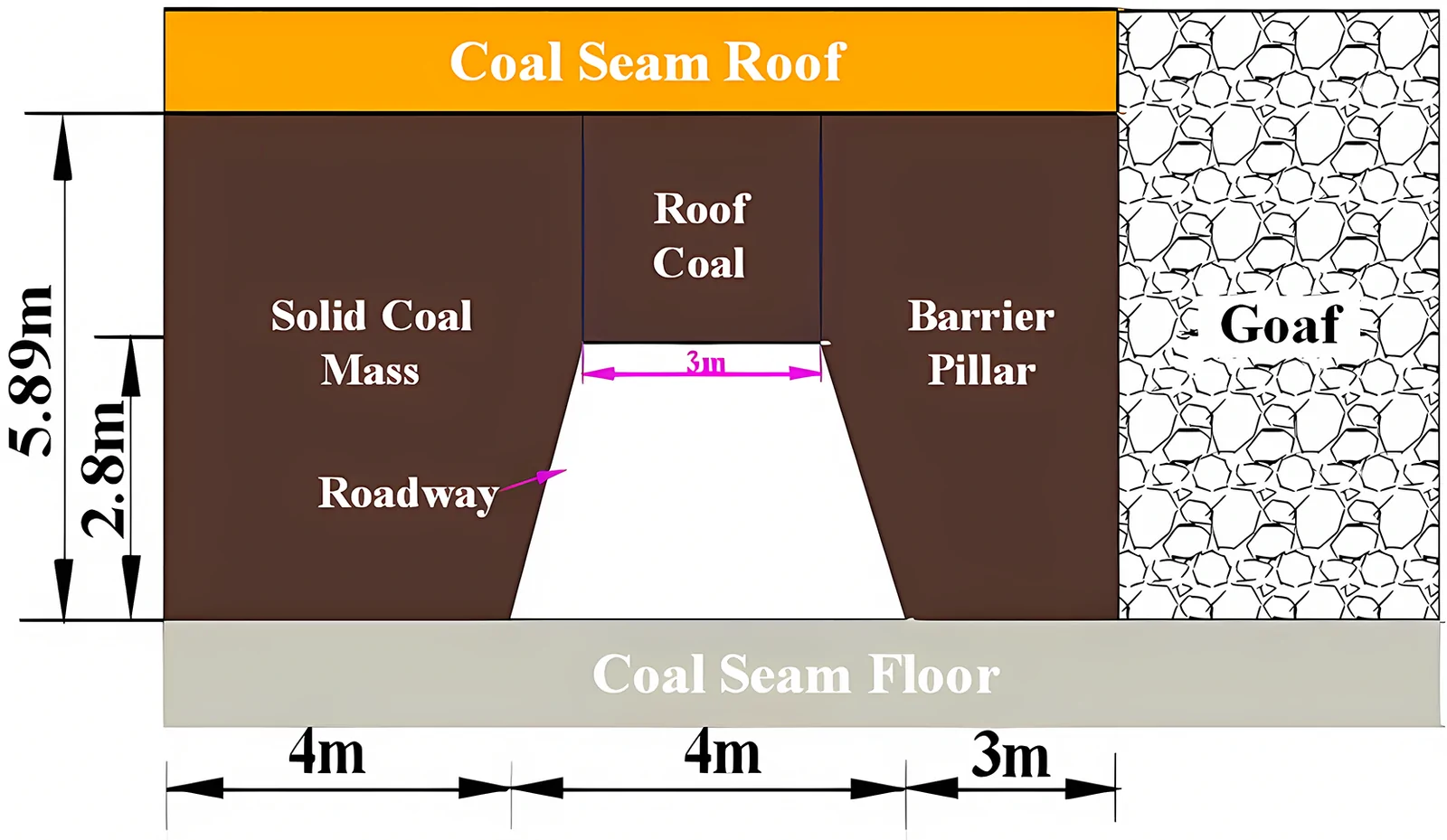
Field geometry of the 16035 fully mechanized top-coal caving face.

**Fig 3:**
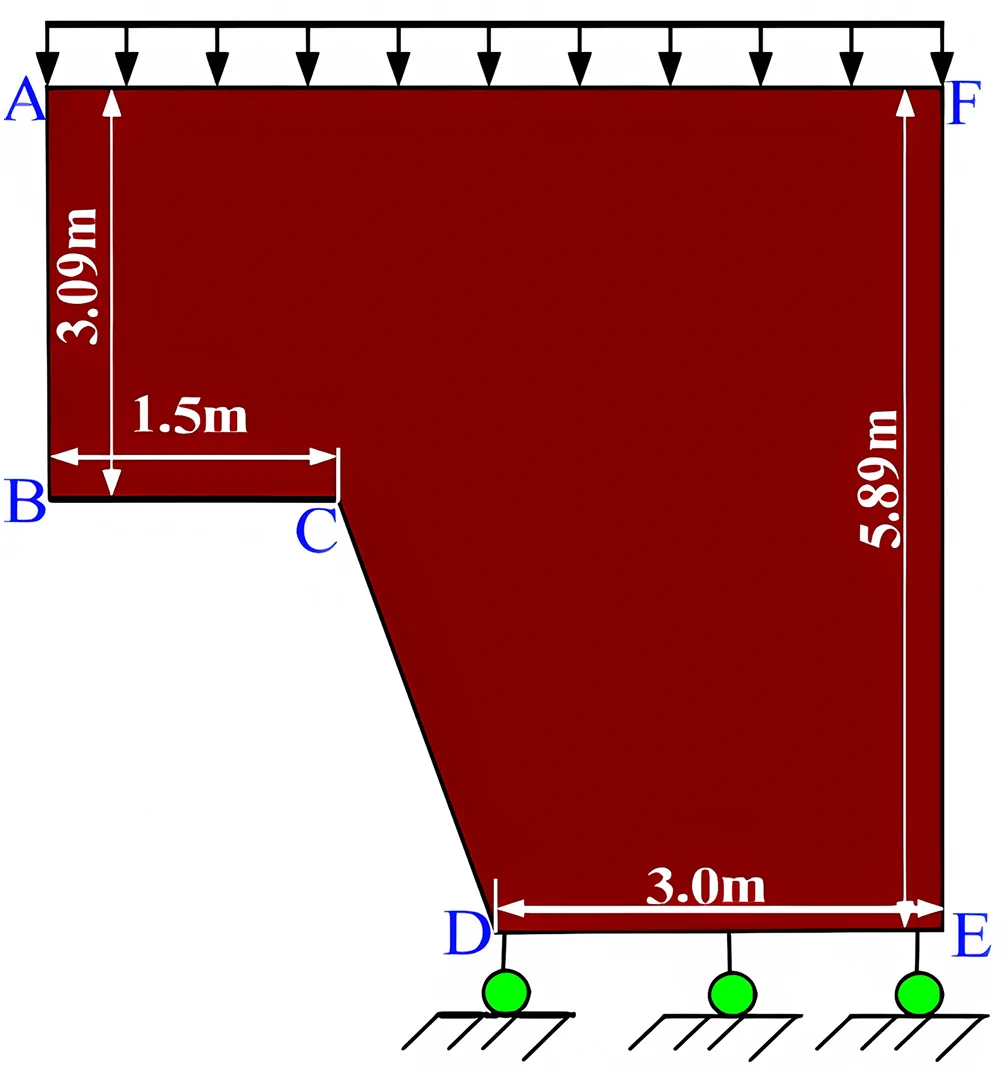
Model domain of the 16035 fully mechanized top-coal caving face.

The 16035 fully mechanized top-coal caving face in the Liupanshui mining area was selected to examine the reasonableness of the coupled model under field conditions. The coal seam at this face is of medium rank and has experienced obvious gas intrusion during mining. The mining thickness was 5.89 m, the cutting height was 2.8 m, the dip length of the face was 202.8 m, and the strike length was 2162.6 m, with a 3 m coal pillar left in place. Under the influence of mining-induced stress, the coal pillar was fractured and connected with the adjacent goaf. As a result, air leakage and oxygen supply occurred along the gob-side roadway, and spontaneous heating was repeatedly observed in the loose coal near the roadway. Field records showed that the shortest spontaneous-heating period during roadway excavation was 23 days. The field geometry and the corresponding numerical model are shown in Figs 2 and 3, and the model parameters used in this section are listed in Table 2.

**Table 2:** Parameter settings for model validation.

Parameter	*E*	$E_{s}$	υ	$\rho_{s}$	$\rho_{g}$	$\varphi_{0}$	$k_{0}$
Value	2213	6139	0.339	1250	1.213	0.3	$5.0\times 10^{-11}$
Unit	MPa	MPa	——	$\text{kg}/{\text{m}}^{\text{3}}$	$\text{kg}/{\text{m}}^{\text{3}}$	${\text{m}}^{\text{2}}$	${\text{m}}^{\text{2}}$
Parameter	*D*	$Q_{s}$	$Q_{H}$	a	$c_{O_{\text{2}}}^{\text{0}}$	R	$h_{T}$
Value	$2\times 10^{-5}$	0	330.3	0.0235	9.375	8.3143	180
Unit	${\text{m}}^{\text{2}}/\text{s}$	$\text{kg}/{\text{(m}}^{\text{3}}\cdot \text{s})$	kJ/mol	${\mathbb{C}}^{-1}$	$\text{mol}/{\text{m}}^{\text{3}}$	J/(mol·K)	$\text{W}/(m^{\text{2}}\cdot K)$
Parameter	μ	$\gamma_{\text{0}}$	$\alpha_{T}$	$\overset{―}{\kappa_{\text{s}}}$	$\overset{―}{\kappa_{\text{g}}}$	$c{\;}_{\text{ps}}\;$	$c{\;}_{\text{pg}}\;$
Value	$1.84\times 10^{-5}$	0.68	$2.4\times 10^{-5}$	0.12	0.026	1530	2276
Unit	$\text{N}\cdot S/{\text{m}}^{\text{2}}$	$\text{mol}/{\text{(m}}^{\text{3}}\cdot \text{h})$	$K^{-1}$	J/(m·s·K)	J/(m·s·K)	J/(kg·K)	J/(kg·K)

**Fig 4:**
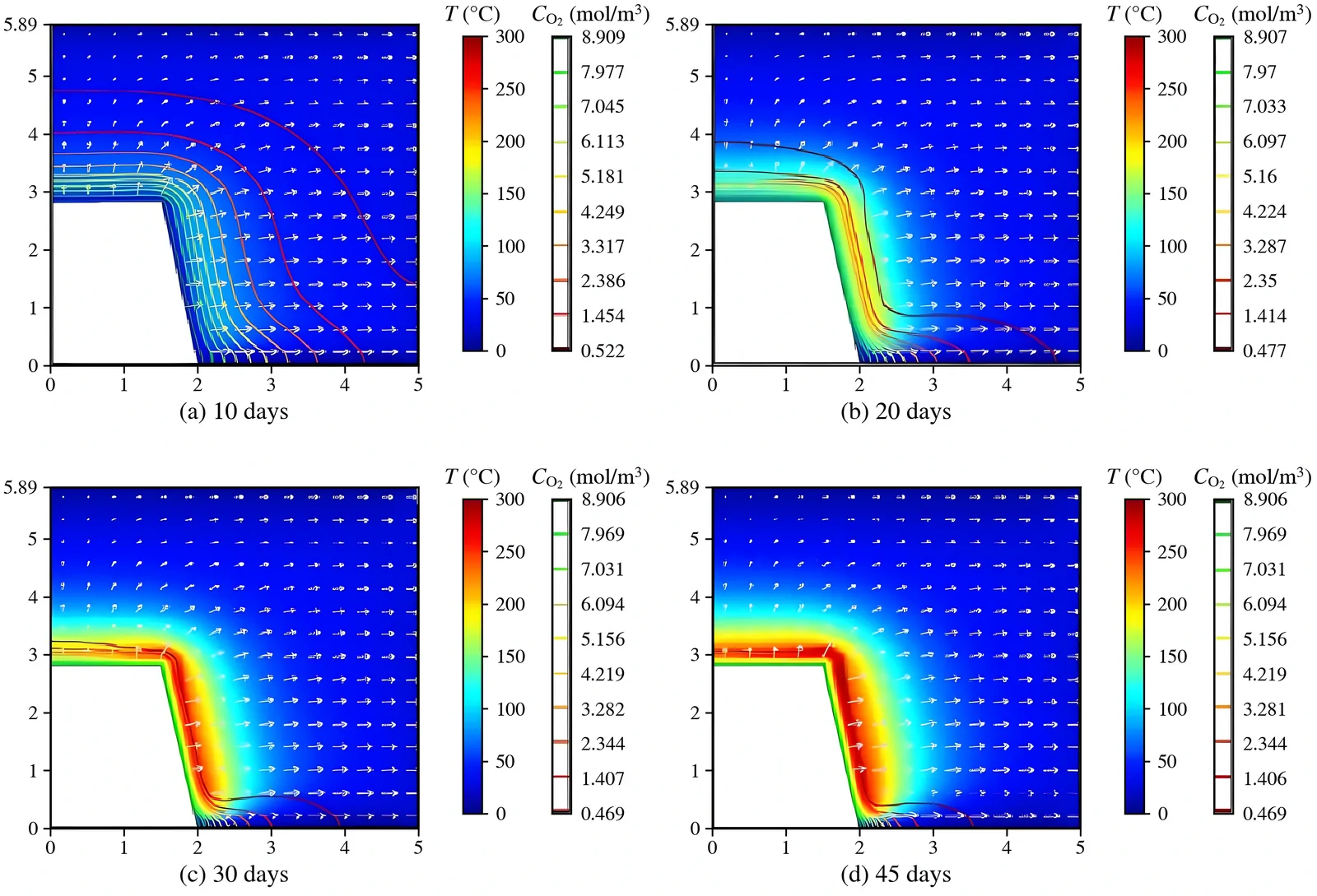
Model validation based on the temporal and spatial evolution of temperature and oxygen concentration near the gob-side roadway at 10, 20, 30, and 45d.

Using the coupled model described in Section 3, the temporal and spatial distributions of temperature and oxygen concentration in the coal near the gob-side roadway were simulated at 10, 20, 30, and 45 days, as shown in Fig 4. In Fig 4, the colored background represents the temperature field, the contour lines represent oxygen concentration, and the arrows indicate the local flow direction near the gob-side roadway. The temperature and oxygen concentration are given in °C and mol/m^3^, respectively.

The results show that, with increasing exposure time, the coal temperature gradually increases and oxygen is consumed more rapidly. In the coal pillar close to the roadway wall, oxygen is depleted quickly in the high-temperature zone. Taking 200°C as the characteristic temperature for spontaneous combustion in this mine, the simulated maximum temperatures at 10 and 15 days are 86.5°C and 267.5°C, respectively. The predicted spontaneous-heating period along the gob-side roadway is 22.7 days, which is close to the shortest field-observed period of 23 days. In addition, after 45 days, the farthest distance from the roadway wall to the high-temperature zone reaches about 0.6 m in the simulation, which is within the field-observed range of 0.5–1.5 m for spontaneous-heating occurrence. These comparisons indicate that the model can reasonably reproduce the main features of thermal evolution and oxygen consumption in fractured coal under high-temperature field conditions. Therefore, this comparison supports the thermal-gas coupling treatment and the field parameter setting used in the model. However, because the main purpose of this study is to analyse gas migration, an additional validation of gas-seepage behavior is required and is presented in Section 4.2.

### 4.2. Validation of gas-extraction seepage behaviour at field site

To further examine the gas-seepage prediction capability of the proposed rough-fracture multi-field coupled model, an additional field comparison was conducted using gas-production data from the Horseshoe Canyon coalbed methane well [35]. The validation in Section 4.1 mainly evaluates the thermal response of fractured coal near the roadway, while this independent case focuses on the seepage response during gas extraction. The main coal-seam and methane parameters used in the simulation are listed in Table 3, including coal porosity, burial depth, Biot coefficient, boundary pressure, wellbore production pressure, fracture porosity, coal permeability, coal compressibility, and elastic parameters.

**Table 3:** Coal-seam and methane parameters used for the independent field validation [35].

Parameter	Value	Unit
Coal porosity	1.1	%
Fracture porosity	0.1	%
Burial depth	500	m
Coal density	1500	kg/m³
Biot coefficient	0.66	/
Lateral stress coefficient	0.667	/
Overburden stress gradient	22.15	kPa/m
Boundary pressure	4.83	MPa
Initial water pressure	4.83	MPa
Wellbore production pressure	1.41-3.42	MPa
Coal permeability	50.1	mD
Coal Young’s modulus	3950	MPa
Coal Poisson’s ratio	0.4	/

The field production data do not provide a complete three-dimensional well pattern and local fracture geometry. Therefore, the gas-extraction process was represented by an equivalent single-well drainage model [35]. The reservoir was simplified as a two-dimensional axisymmetric cylindrical domain, with the production well located at the inner boundary and the far-field drainage boundary located at the outer radius. According to the reported gas-in-place and coal-seam parameters, the coal-seam thickness was set to 8.99m. The model domain was discretized by finite elements, with a denser mesh near the wellbore to capture the larger pressure gradient during early gas extraction.

The initial gas pressure in the coal seam was set according to the reported reservoir pressure, and the initial stress state was determined from the burial depth and overburden stress gradient. The inner wellbore boundary was assigned a pressure-controlled production condition, with the wellbore pressure varying within the reported range of 1.41–3.42MPa. The gas production rate was not prescribed, but was calculated from the pressure gradient and gas-flow response at the wellbore boundary. The outer radial boundary was treated as a closed drainage boundary for gas flow, representing the finite drainage area of a single production well. The upper and lower boundaries were set as no-flow boundaries. For the mechanical field, the top boundary was loaded by the overburden stress, the bottom boundary was fixed in the vertical direction, and the outer radial boundary was constrained in the normal direction to represent lateral confinement.

**Fig 5:**
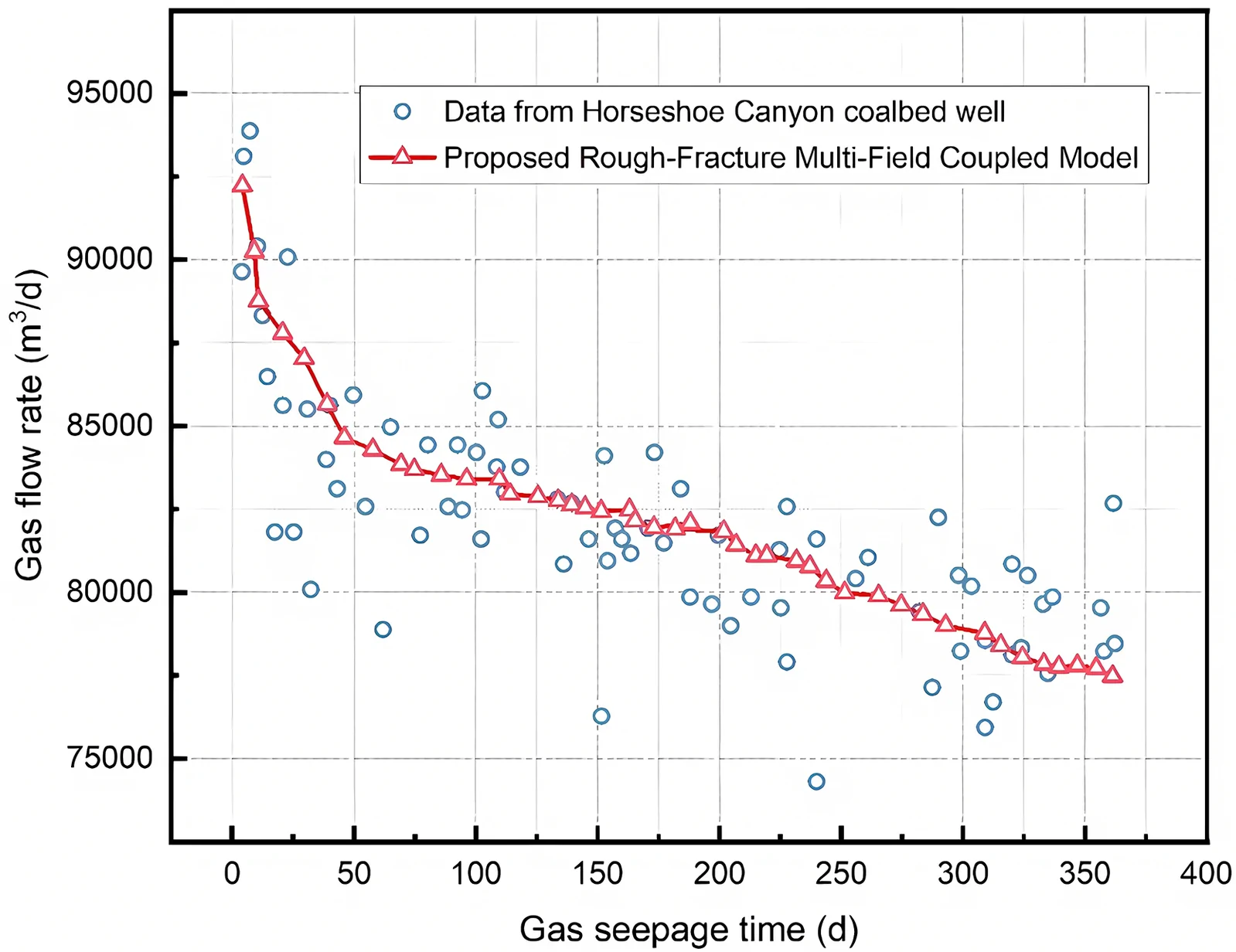
Comparison between simulated and field-measured gas-production rates from the Horseshoe Canyon coalbed methane well.

Fig 5 compares the simulated gas-production rate with the field data [35]. The measured production rate shows an overall decreasing trend during the production period, with local scatter caused by field operation and geological variability. The simulated curve reproduces this main decline. The gas-production rate decreases from 9.22 × 10^4^m^3^/d at the early stage to 7.75 × 10^4^m^3^/d after about 360d. The decline is relatively rapid during the early production period and then becomes slower, indicating a transition from initial pressure depletion to a later gas-supply process controlled by matrix-fracture exchange and coal-seam seepage.

A quantitative comparison was also conducted. Because the sampling times of the field data and the simulated curve were not identical, the simulated values were interpolated to the field measurement times. All field points within the simulation period were included in the error calculation. The comparison gives an RMSE of 2.28 × 10^3^m^3^/d, an MAE of 1.70 × 10^3^m^3^/d, and an MRE of 2.09%. The normalized RMSE is about 2.78% of the mean measured production rate. These results indicate that the model can reproduce the average gas-extraction seepage behavior with acceptable accuracy.

The remaining deviations mainly come from short-term fluctuations in the field data. Field gas production can be affected by wellbore pressure adjustment, monitoring uncertainty, local geological heterogeneity, and operational disturbance. The numerical model represents the average seepage response under the measured coal-seam parameters and does not reproduce every local fluctuation in the production record. Nevertheless, the simulated curve remains within the main distribution range of the field data and captures the long-term decline of gas production. This comparison provides supplementary validation for the gas-extraction seepage component of the proposed model and supports its use in the subsequent analysis of gas migration in fractured coal seams.

## 5. Gas migration and coal-rock response in the 16035 working face

After the coupled thermal response and gas-extraction seepage behavior of the model had been examined, the model was used to study gas migration and coal-rock response in the 16035 working face of the Liupanshui mining area. According to the geological survey described in Section 2, multiple fracture zones are developed in this working face. A near-roadway domain of 5 m × 5 m was therefore selected to examine gas seepage, temperature change, and coal-rock stability under high-temperature conditions. In the model, the right boundary represents the roadway side, where the temperature is lower and gas is absent, whereas the left boundary represents the coal seam, where both gas pressure and temperature are higher. The fracture network in the model was generated by the method described in Section 3.

### 5.1 Effect of fracture zones on gas migration and coal-rock response

**Fig 6:**
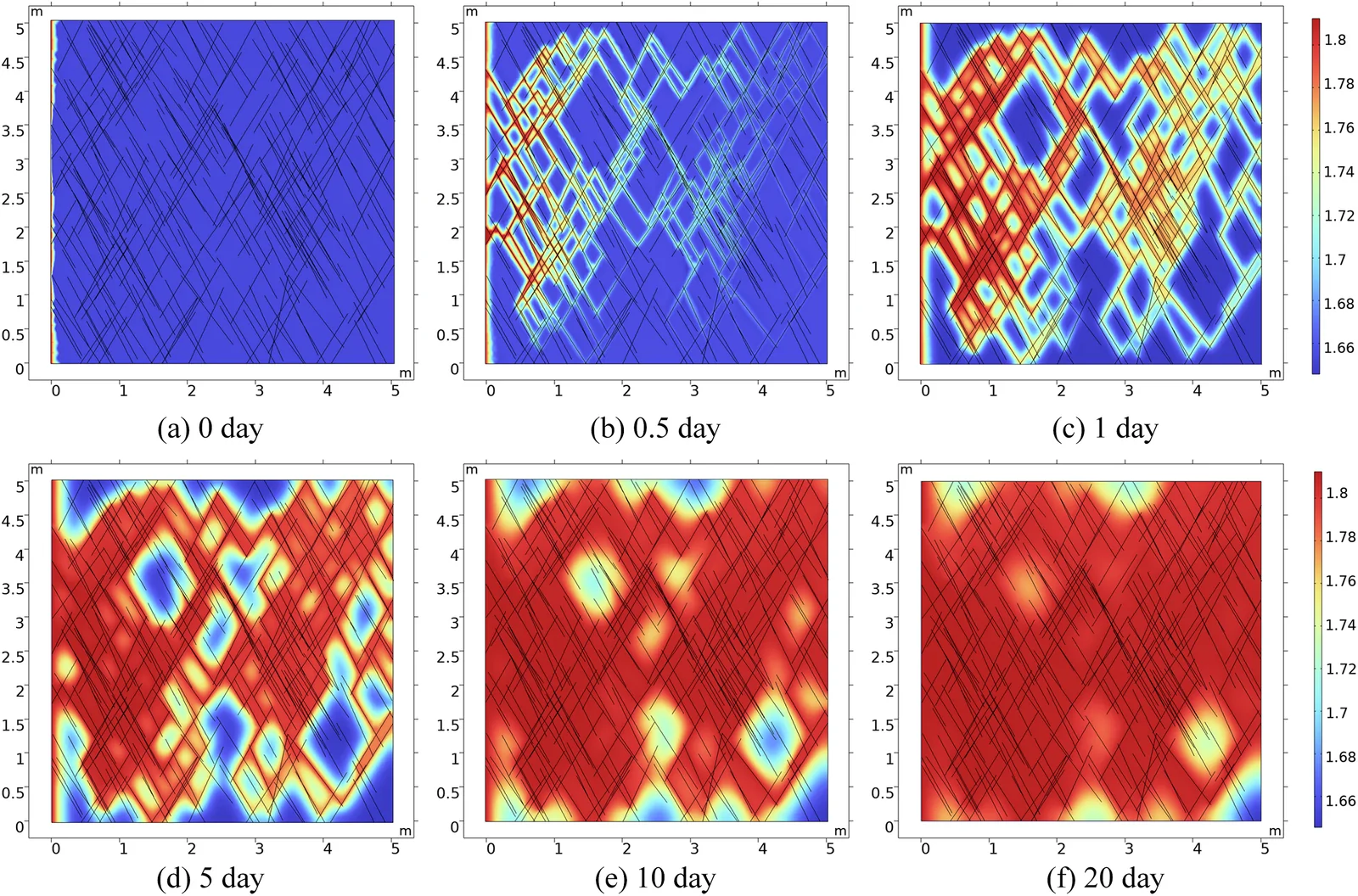
Temporal and spatial evolution of gas pressure in the high-temperature coal-rock mass with fracture zones (MPa).

**Fig 7:**
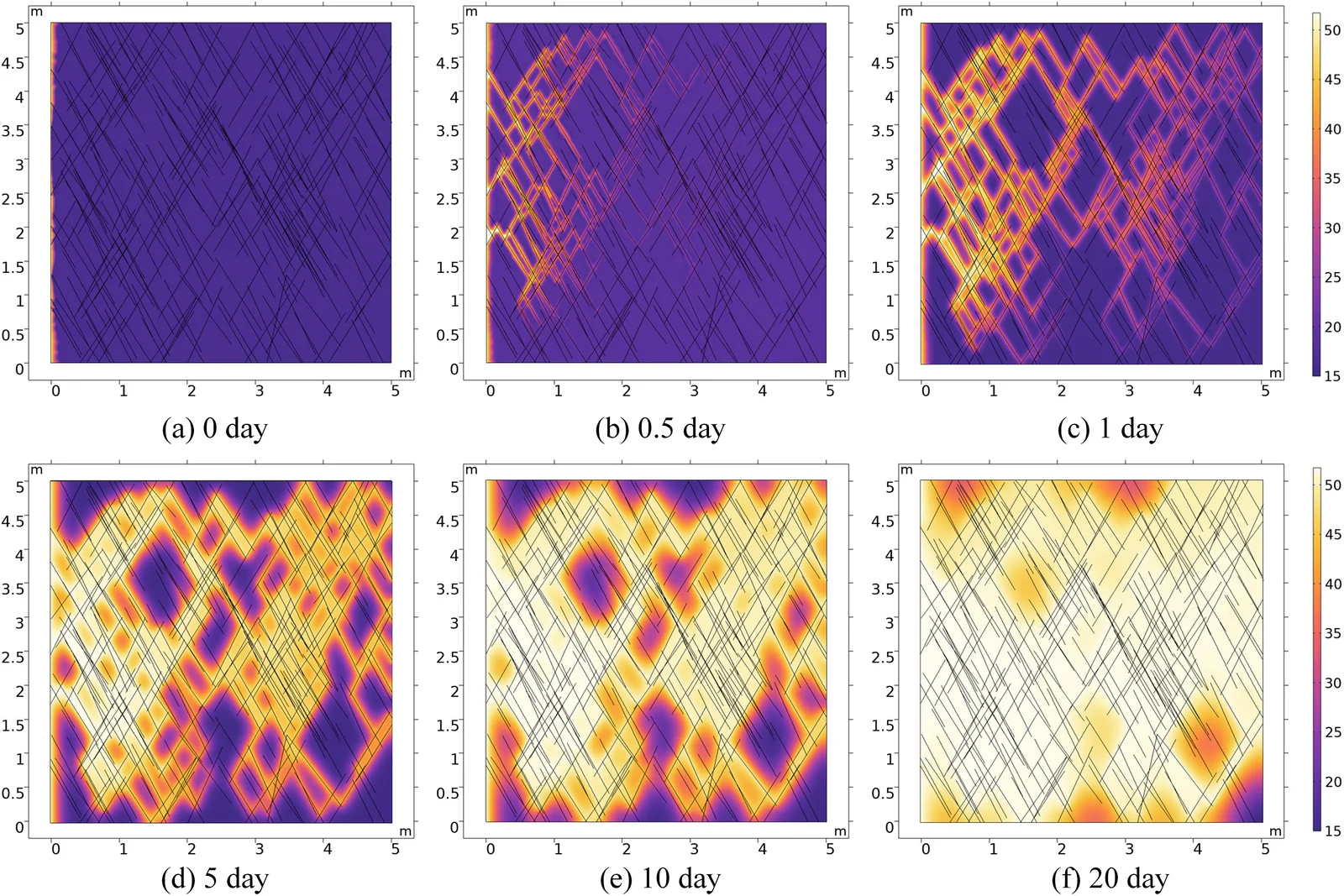
Temporal and spatial evolution of temperature in the high-temperature coal-rock mass with fracture zones (°C).

**Fig 8:**
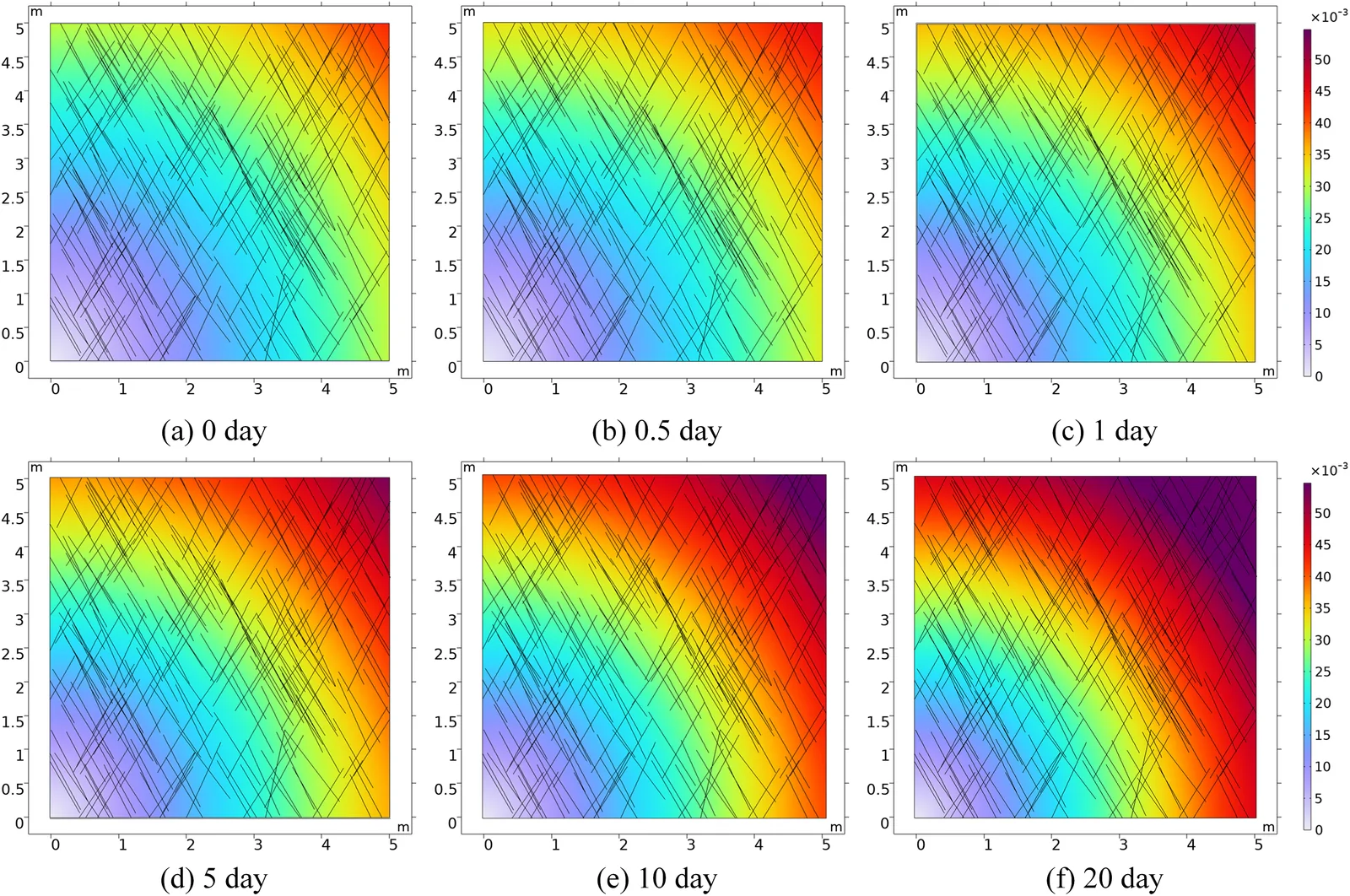
Temporal and spatial evolution of displacement in the high-temperature coal-rock mass with fracture zones (m).

The effects of fracture zones on gas pressure, temperature, stress, and displacement during gas migration were examined at different intrusion times. In Figs 6–8, the color contours represent gas pressure, temperature, and displacement, respectively, while the black line segments denote the generated fracture network. The model coordinates are given in m, and the corresponding field variables are given in MPa, °C, and m.

Fig 6 shows the temporal and spatial evolution of gas pressure in the high-temperature coal-rock mass with fracture zones. At 0d, the gas pressure in most of the 5m × 5m domain remains close to the low-pressure level of about 1.65–1.66MPa, and only the coal-seam side shows a higher pressure boundary. After 0.5d, the gas pressure begins to increase rapidly along connected fracture bands, and local high-pressure zones above 1.72MPa appear in the internal coal-rock mass. After 1d, the high-pressure region further extends along the fracture network, and several connected fracture paths reach 1.76–1.80MPa. At 5d, most connected fracture regions have already reached a high-pressure state of about 1.78–1.80MPa. From 10 to 20d, the pressure field becomes more uniform, although several isolated low-pressure blocks remain in poorly connected regions.

These results show that fracture zones do not only accelerate gas migration, but also control the spatial position of pressure accumulation. Compared with intact coal-rock regions, connected fractures provide low-resistance channels for pressure transfer, so the pressure front can move from the coal-seam side toward the roadway side within the first 0.5-1d. The pressure difference between connected fractures and isolated matrix blocks is about 0.05–0.10MPa during the early stage, indicating that fracture connectivity directly controls local gas accumulation near the roadway.

Fig 7 shows the temperature evolution during gas migration. The temperature field changes from a boundary-controlled distribution to a fracture-controlled distribution as gas migration proceeds. At 0d, most of the domain remains at a low-temperature level of about 15–20°C, except for the high-temperature coal-seam boundary. After 0.5d, local temperature increases to about 35–45°C along several connected fracture paths. After 1d, the high-temperature branches become more continuous, and some fracture-controlled regions reach above 45°C. At 5d, the high-temperature zone expands to most connected fracture regions, while several isolated blocks still remain below 35°C. At 10-20d, the temperature field becomes more uniform, and most of the domain approaches the upper temperature range of 45–50°C.

The temperature evolution is consistent with the gas-pressure distribution in Fig 6. This consistency indicates that gas migration is accompanied by heat transfer, and that the fracture network provides preferential channels for both pressure propagation and heat transport. The temperature difference between fracture-controlled high-temperature zones and isolated low-temperature blocks reaches about 10–20°C during the first 1-5d. This difference produces non-uniform thermal expansion in the coal-rock mass and becomes an important source of later deformation.

Fig 8 shows the displacement field during gas migration. The displacement distribution presents a clear spatial gradient from the lower-left part of the model to the upper-right part near the roadway roof. At the early stage, the displacement field is mainly controlled by the mechanical boundary condition and the initial stress state. With continued gas migration and heat transfer, the high-displacement region expands gradually. The region with displacement larger than 40 × 10 − 3m is mainly limited to the upper-right part at 0-1d, but it expands toward the internal coal-rock mass after 5d. At 20d, the maximum displacement reaches the upper range of the contour scale, about 50 × 10 − 3m, and the high-displacement zone becomes more continuous near the roadway side.

This result indicates that coal-rock deformation has a delayed response compared with gas-pressure transfer and heat transfer. In the first 0.5-1d, gas pressure and temperature already migrate rapidly along the fracture network, whereas the displacement field changes more gradually. After 5d, the accumulated pressure disturbance and thermal expansion jointly increase coal-rock deformation. Therefore, the fracture network affects coal-rock stability through two linked processes: rapid gas and heat migration in the early stage, followed by non-uniform deformation during continued intrusion.

**Fig 9:**
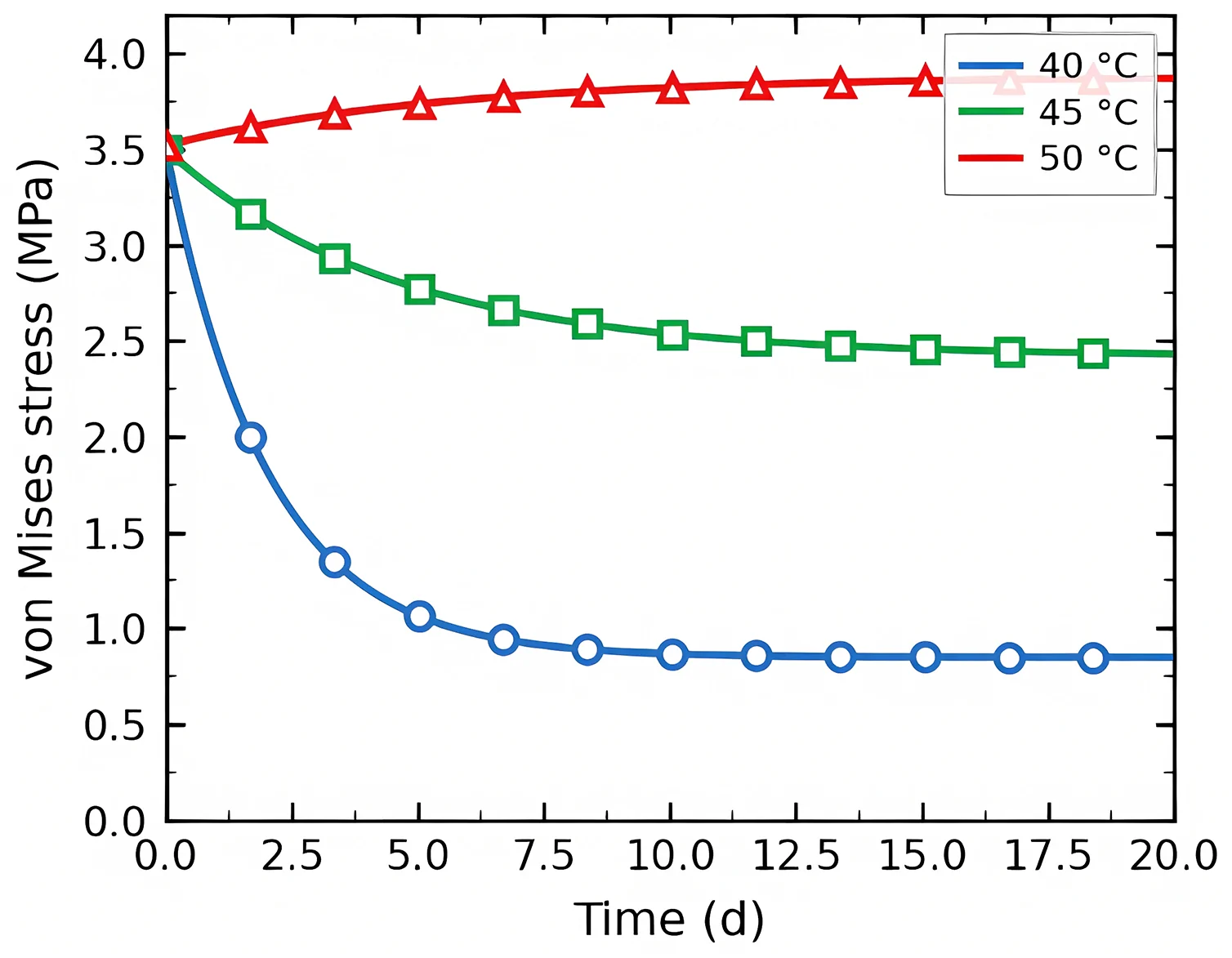
Evolution of stress in the coal-rock mass with fracture zones under different initial temperatures.

Because the Liupanshui mining area is a high-temperature mine, the effect of initial coal-seam temperature on gas migration and coal-rock stability was further examined. Fig 9 shows the evolution of von Mises stress under initial coal-seam temperatures of 40°C, 45°C, and 50°C. At 40°C, the local von Mises stress decreases rapidly during the first 6-8d and then becomes nearly stable. At 45°C, the stress decrease is weaker and the final stress remains higher than that in the 40°C case. At 50°C, the stress does not decrease. Instead, it increases gradually with time. This result indicates that the early stress decrease is not a universal response of the whole coal-rock mass. It is a local and stage-dependent stress perturbation caused by gas-pressure redistribution under lower-temperature conditions. Because the magnitude of this perturbation is limited relative to the total in-situ stress level, it should not be interpreted as large-scale stress release. The more important trend is the change in the controlling mechanism: gas-pressure redistribution dominates the early local unloading at lower temperature, whereas thermal expansion becomes dominant at higher temperature and offsets or exceeds the unloading effect caused by gas seepage.

**Fig 10:**
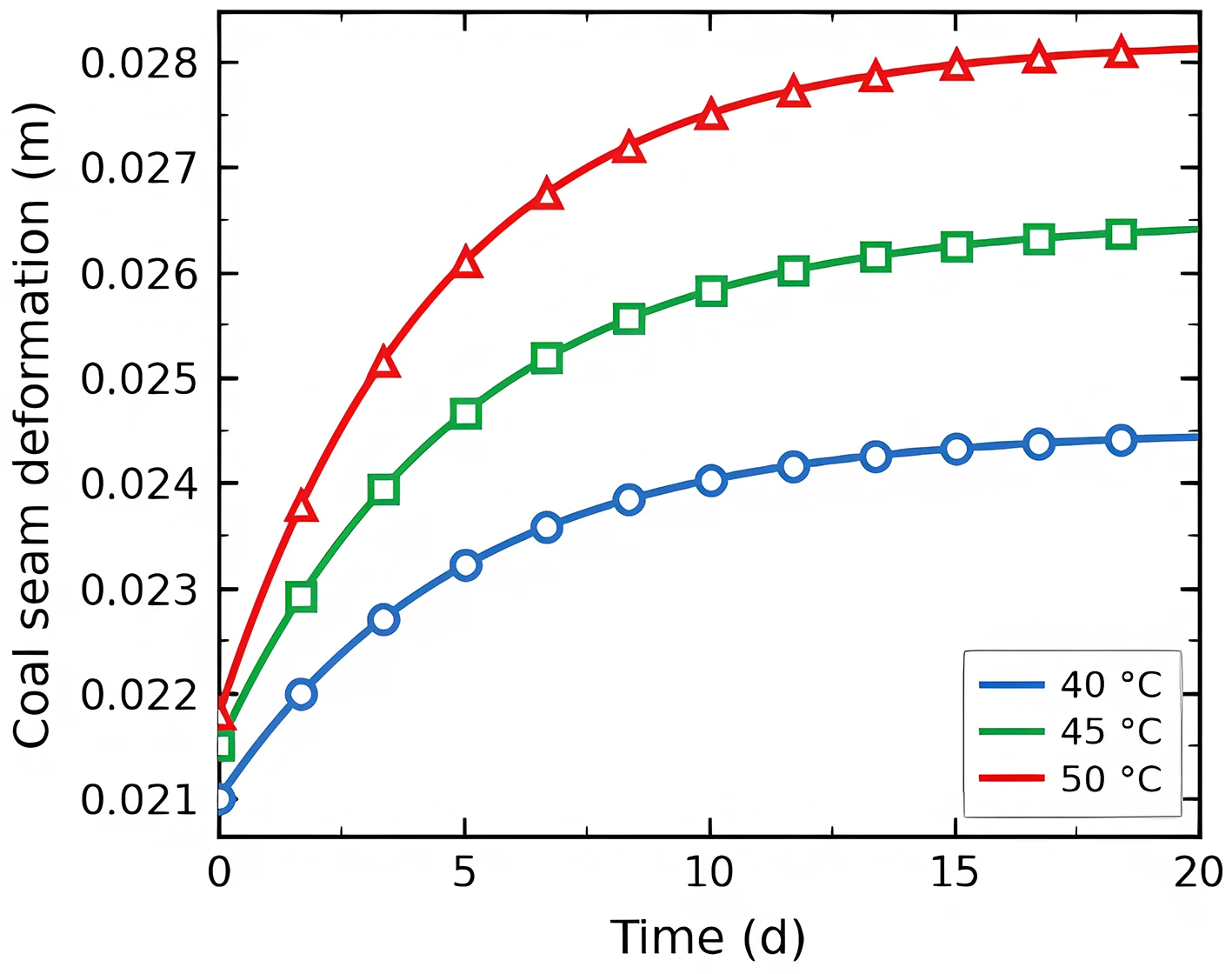
Displacement evolution in the coal-rock mass with fracture zones under different initial temperatures.

Fig 10 shows the corresponding displacement evolution. The displacement increases rapidly during the first 5-8d and then tends to stabilize. At 20d, the maximum displacement is about 0.0244m, 0.0264m, and 0.0282m for initial temperatures of 40°C, 45°C, and 50°C, respectively. Compared with the 40°C case, the final displacement increases by about 8.2% at 45°C and by 15.8% at 50°C. This result confirms that higher initial coal-seam temperature reduces coal-rock stability by increasing thermal expansion and enlarging the deformation zone near the roadway. The rapid displacement growth in the first several days also agrees with the pressure and temperature propagation shown in Figs 6 and 7.

**Fig 11:**
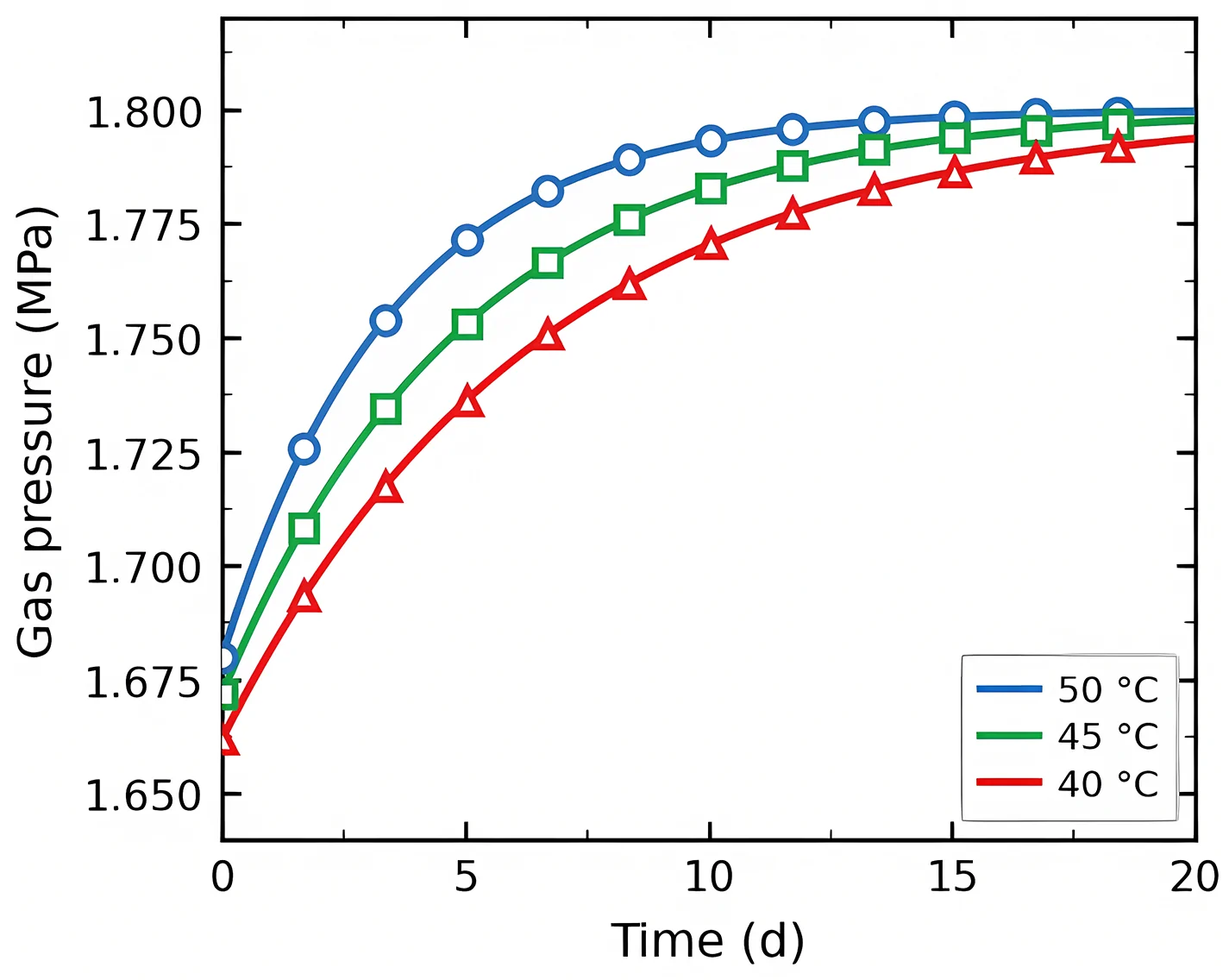
Evolution of gas pressure in the coal-rock mass with fracture zones under different initial temperatures.

Fig 11 shows the gas-pressure evolution near the roadway under different initial coal-seam temperatures. In all temperature cases, gas pressure increases rapidly during the first 5d and then approaches a stable value. The pressure increases from about 1.66–1.68MPa at the initial stage to about 1.79–1.80MPa at 20d. Higher initial temperature leads to higher gas pressure throughout the migration process. The pressure difference between the 50°C and 40°C cases is most obvious during the early stage, especially within the first 3-8d, and then decreases as the pressure field approaches equilibrium. When the initial temperature increases from 40°C to 50°C, the maximum gas pressure increases by 4.3%.

The temperature effect on gas pressure is controlled by two opposite mechanisms. Higher temperature promotes methane desorption and increases gas migration from the coal matrix to fractures. At the same time, thermal expansion compresses part of the fracture space and tends to reduce local permeability. In the present case, the desorption-enhancement effect is stronger than the permeability-reduction effect, so the overall gas pressure near the roadway still increases with initial coal-seam temperature. This result indicates that gas hazard assessment in high-temperature fractured coal seams should consider not only the initial gas pressure, but also the coupled effect of temperature-driven desorption, fracture-controlled migration, and thermal deformation.

### 5.2 Effect of fracture roughness on permeability and gas desorption

**Fig 12:**
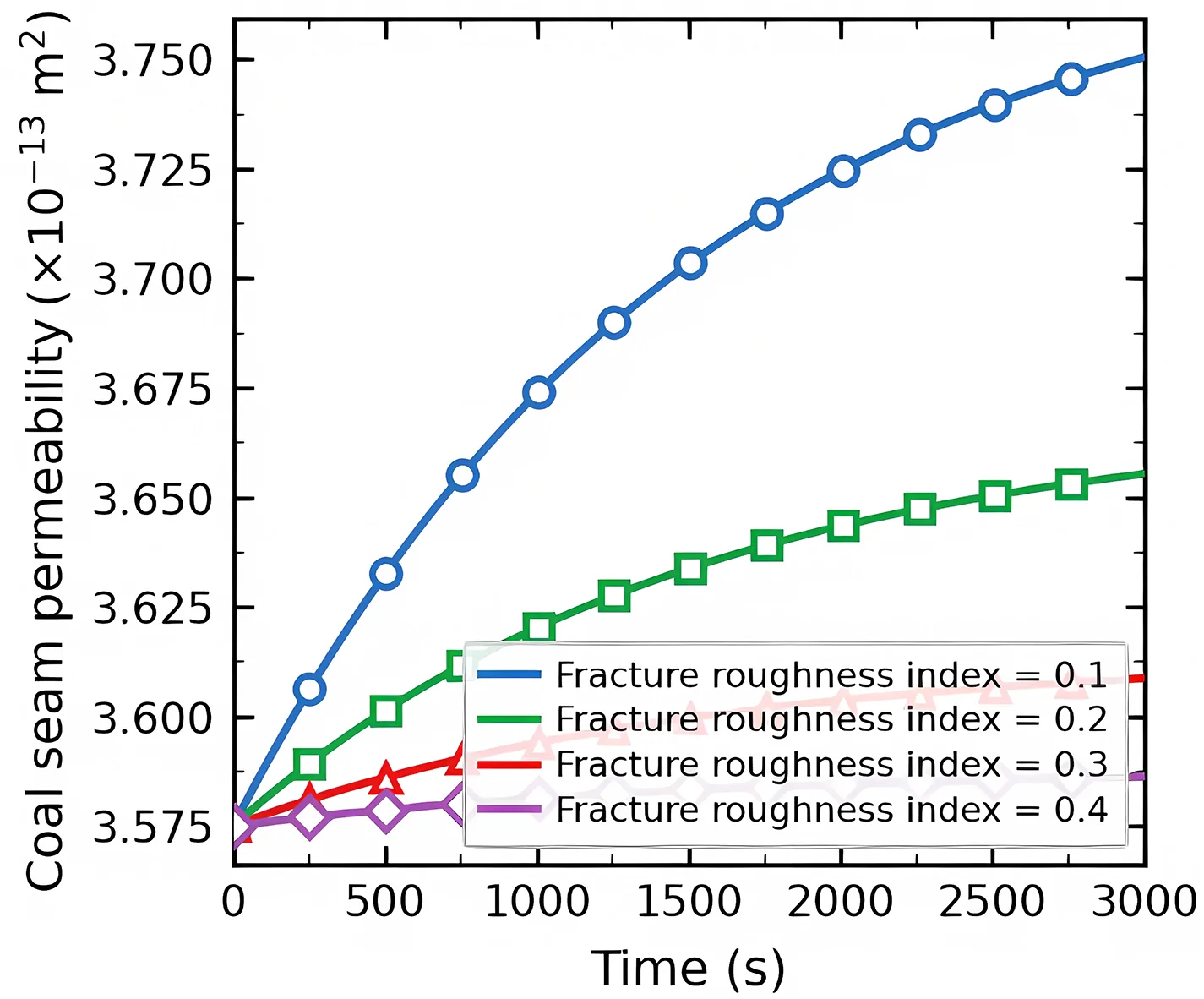
Effect of fracture roughness index on coal-seam permeability.

**Fig 13:**
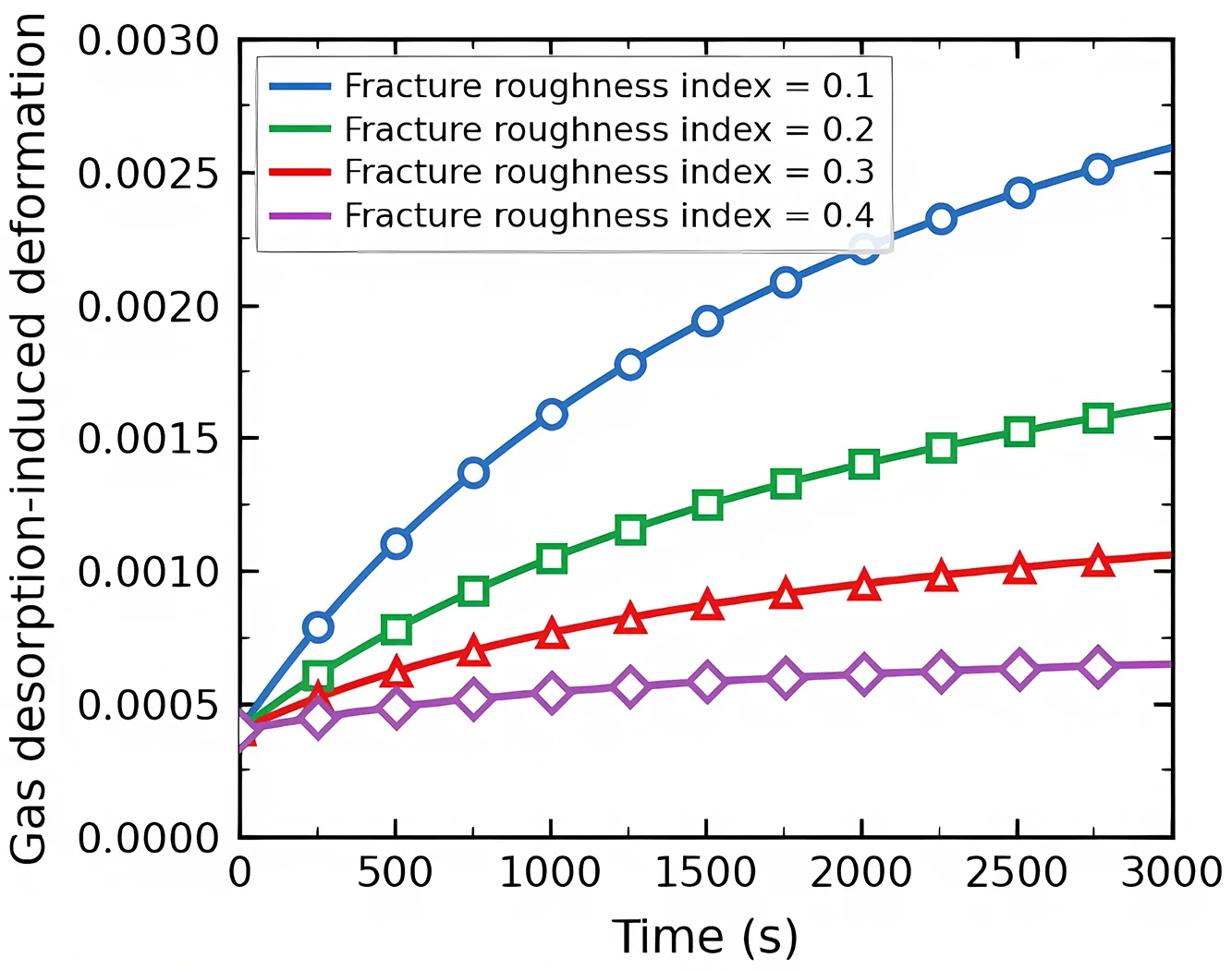
Effect of fracture roughness index on gas desorption at the working face.

The effect of fracture roughness on gas transport in the 16035 working face was examined under coupled thermal, seepage, and deformation conditions, as shown in Figs 12 and 13. Fig 12 shows the permeability evolution under different fracture roughness indices. Different curves correspond to controlled fracture roughness indices. A smaller χ denotes a smoother fracture surface, whereas a larger χ denotes stronger asperity obstruction.

For all roughness conditions, permeability increases with gas-intrusion time, but the growth rate gradually decreases after about 1500s. When *χ* = 0.1, the coal-seam permeability increases from about 3.58 × 10^-13^m^2^ at the initial time to about 3.75 × 10^-13^m^2^ at 3000s. When χ = 0.4, the permeability only shows a slight increase and remains at a lower level. At the same time, the permeability follows the order *χ* = 0.1 > *χ* = 0.2 > *χ* = 0.3 > *χ* = 0.4 during the whole migration process. When the fracture roughness index decreases from 0.4 to 0.1, the maximum permeability increases by 6.18%.

This result indicates that fracture roughness mainly changes gas transport by modifying the equivalent hydraulic aperture of the fracture. A smaller *χ* represents a smoother fracture surface, a smaller contact obstruction, and a larger effective flow space. Therefore, gas can pass through the fracture network more easily. However, the permeability variation remains relatively limited in the simulated roughness range, because permeability is controlled by the equivalent flow channel and does not increase without limit when the fracture surface becomes smoother.

Fig 13 shows the evolution of gas desorption intensity under different fracture roughness conditions. The desorption intensity increases with time for all roughness cases, but the increase is much more pronounced under lower roughness. At 3000s, the desorption intensity reaches about 2.5 × 10^−3^ for *χ* = 0.1, whereas it is only about 6.0 × 10^−4^ to 6.5 × 10^−4^ for *χ* = *0*.4. The relative difference between the two cases reaches 75.7% when the *χ* = 0.1 result is used as the reference. This means that the desorption response is much more sensitive to fracture roughness than the permeability response.

The difference between the permeability increase and the desorption increase can be explained by their different physical meanings. Permeability describes the instantaneous flow capacity of the equivalent fracture channel, so its variation is mainly controlled by changes in hydraulic aperture. Gas desorption intensity is not controlled by permeability alone. It is also affected by pressure decline in the fracture, matrix-fracture gas exchange, contact area between gas and coal, and the cumulative desorption process. A smoother fracture only produces a moderate increase in permeability, but it allows gas to leave the fracture network more rapidly and maintains a stronger pressure difference between the coal matrix and the fracture. This pressure difference continuously activates methane desorption from the coal matrix. Therefore, a relatively small permeability difference can lead to a much larger difference in desorption intensity over 3000s.

### 5.3. Effect of coal-seam temperature under rough fracture conditions

**Fig 14:**
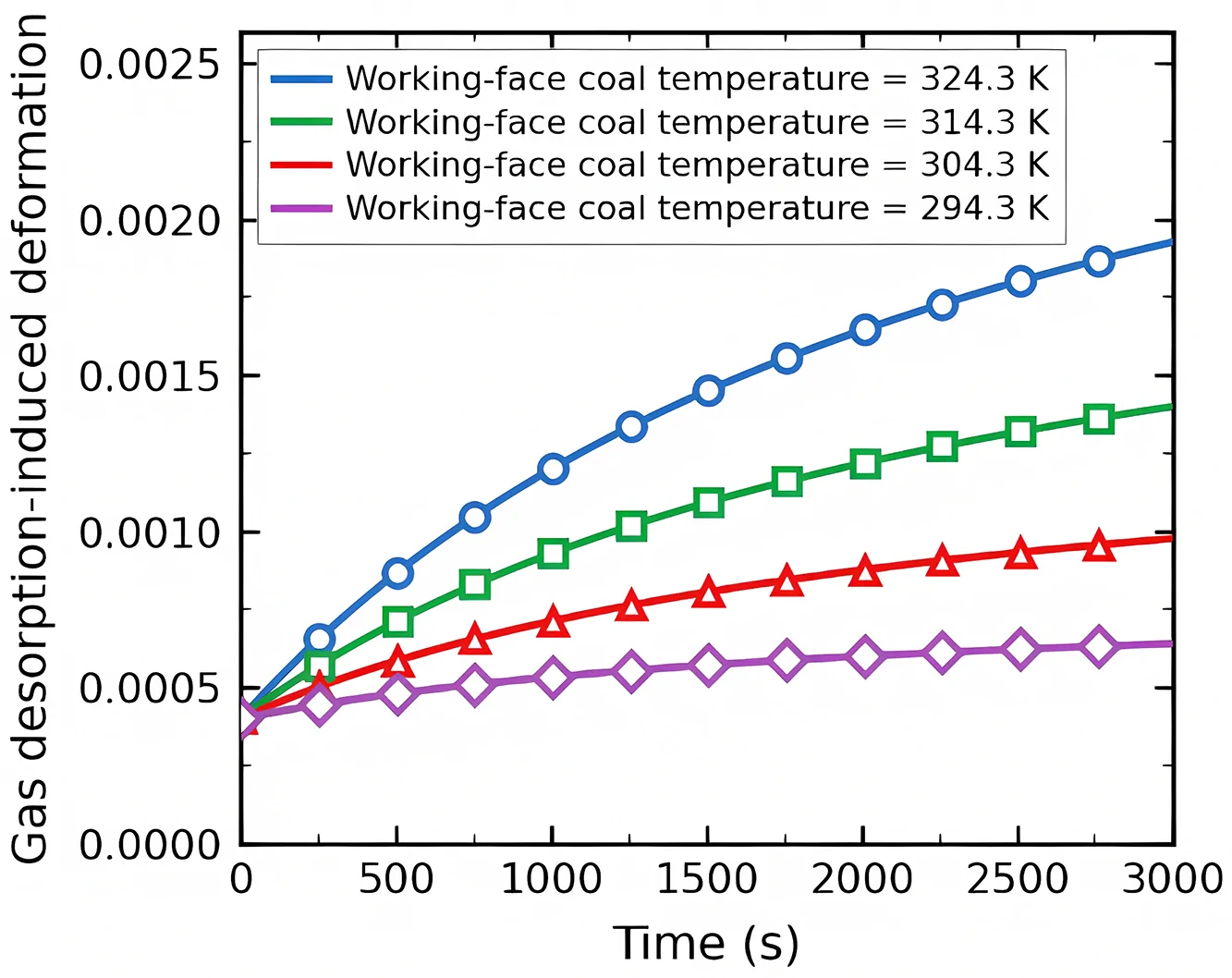
Effect of coal-seam temperature on gas desorption under rough fracture conditions.

**Fig 15:**
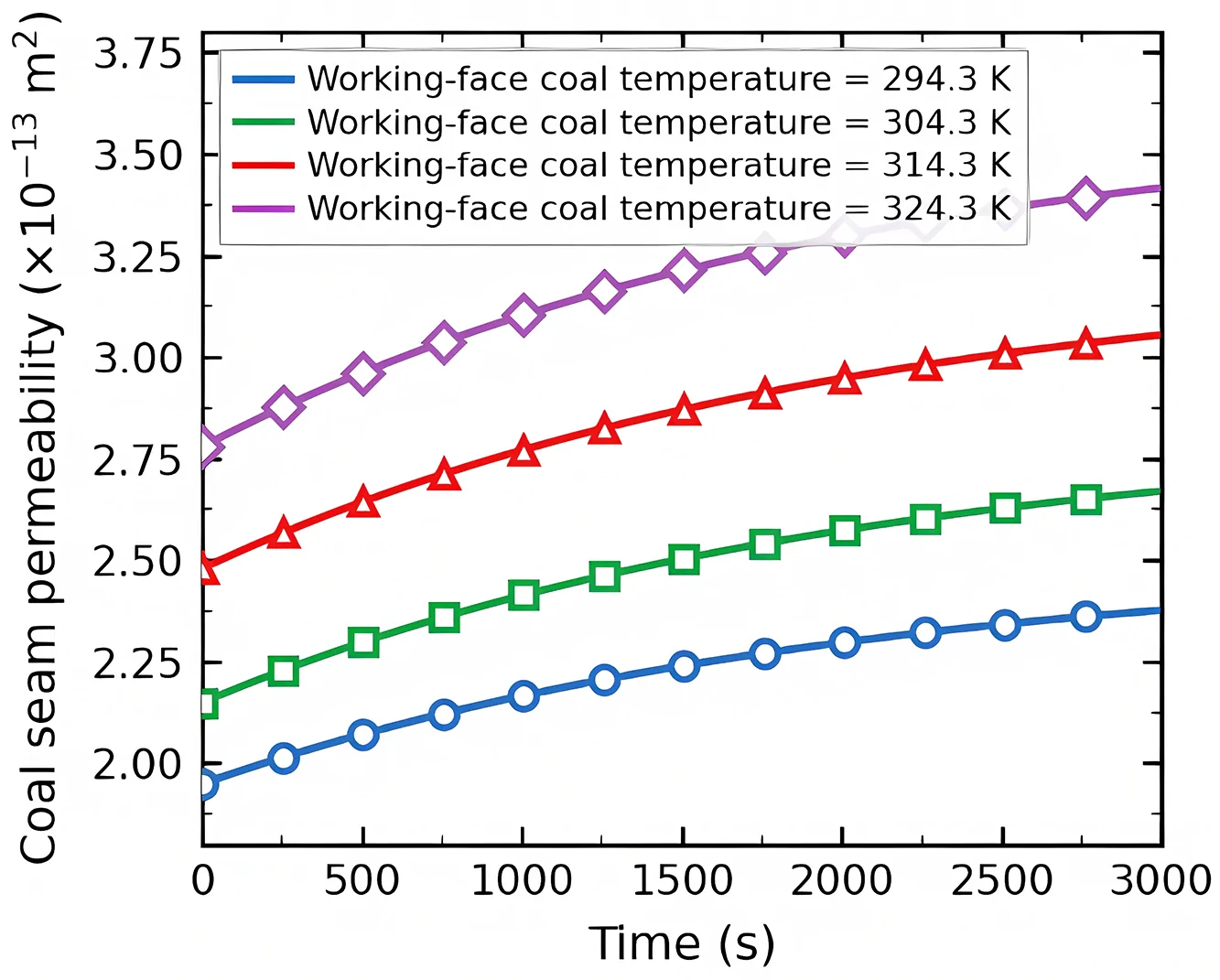
Effect of coal-seam temperature on permeability under rough fracture conditions.

The effect of coal-seam temperature on gas desorption and permeability under rough fracture conditions was further examined, as shown in Figs 14 and 15. Fig 14 shows that gas desorption intensity increases with time under all temperature conditions. The increase is rapid during the first 1000s and then becomes more gradual. At 3000s, the desorption intensity is about 6.0 × 10^−4^ to 6.5 × 10^−4^ at 294.3K, about 1.0 × 10^−3^ at 304.3K, about 1.4 × 10^−3^ at 314.3K, and about 1.9 × 10^−3^ at 324.3K. Thus, when the temperature increases from 294.3K to 324.3K, the desorption intensity at 3000s increases to nearly three times its original value.

This trend is caused by the direct effect of temperature on methane adsorption and desorption in the coal matrix. Higher temperature increases the thermal motion of methane molecules and weakens methane adsorption on coal pore surfaces. As a result, more adsorbed methane changes into free gas and enters the fracture network. Under rough fracture conditions, this temperature-driven desorption is further affected by fracture-controlled gas migration, because connected fractures provide the main pathways for the desorbed gas to move toward the working face.

Fig 15 shows the effect of coal-seam temperature on permeability. In contrast to a simple fracture-closure interpretation, the simulated permeability increases with both time and temperature in the present rough-fracture model. At 3000s, the permeability is about 2.35 × 10^−13^m^2^ at 294.3K, about 2.65 × 10^−13^m^2^ at 304.3K, about 3.05 × 10^−13^m^2^ at 314.3K, and about 3.40 × 10^−13^m^2^ at 324.3K. Compared with the 294.3K case, the permeability at 324.3K increases by about 45%. This temperature-induced permeability difference is larger than the permeability difference caused by fracture roughness in Fig 14, indicating that temperature changes not only methane desorption, but also the coupled deformation and flow state of the coal-rock mass.

The permeability increase under higher temperature is controlled by two competing effects. On the one hand, thermal expansion of the coal-rock mass tends to reduce part of the fracture aperture. On the other hand, higher temperature promotes methane desorption, pressure redistribution, and matrix shrinkage associated with gas release. These processes can increase the effective flow space of the fracture system. In the present simulation range of 294.3-324.3K, the desorption-induced and pressure-driven effects dominate over the thermal-closure effect, so the net permeability response is positive. This explains why both desorption intensity and permeability increase with coal-seam temperature under rough fracture conditions.

Parameter uncertainty is unavoidable in field-scale gas-migration modelling because fracture geometry, aperture, roughness, gas pressure, matrix permeability, and temperature conditions vary in underground coal seams. In this study, these uncertainties mainly affect the predicted magnitudes of gas pressure, permeability, desorption intensity, and displacement, especially in highly connected fracture zones. However, the dominant trends remain stable within the investigated parameter ranges (as shown in Figs 4 and 5). Connected fractures still provide preferential channels for gas and heat transfer, higher coal-seam temperature still promotes gas desorption and deformation, and smoother fractures still enhance matrix-fracture gas exchange. Therefore, the model results should be interpreted as quantitative trend predictions under representative field conditions, rather than as single deterministic values for every geological state.

## 6. Conclusions

A multi-field coupled model was developed to investigate gas migration and coal-rock response in high-temperature coal seams containing rough fractures. The model couples gas seepage, heat transfer, coal-rock deformation, gas desorption, and roughness-dependent fracture permeability. Field comparison and numerical analysis led to the following conclusions.

(1) The model reproduced both the thermal response of fractured coal and the average gas-extraction seepage behavior under field conditions. For the 16035 working face, the predicted spontaneous-heating period was 22.7d, close to the field-observed value of 23d, and the simulated high-temperature zone after 45d extended about 0.6m from the roadway wall, within the observed range of 0.5-1.5m. Besides, the simulated gas-production rate agreed with the field data, with an RMSE of 2.28 × 10^3^m^3^/d, an MAE of 1.70 × 10^3^m^3^/d, an MRE of 2.09%, and a normalized RMSE of 2.78%. These comparisons support the use of the model for subsequent gas-migration analysis in fractured coal seams.
(2) Fracture zones controlled the early migration of gas and heat and caused strong spatial heterogeneity near the roadway. Gas pressure propagated rapidly along connected fractures within the first 0.5-1d, with local pressure reaching about 1.76–1.80MPa after 1d. The temperature field showed a similar fracture-controlled pattern, and local temperature along connected fractures increased to about 35–45°C after 0.5d. The pressure and temperature differences between connected fractures and isolated matrix blocks reached about 0.05–0.10MPa and 10–20°C, respectively, confirming that fracture connectivity governs local gas accumulation and heat transfer.
(3) Initial coal-seam temperature changes the controlling mechanism of stress and deformation evolution. Under lower-temperature conditions, gas-pressure redistribution can cause a local and short-term stress decrease. However, this decrease is limited in magnitude and should be interpreted as a local stress perturbation rather than a large-scale stress release. When the initial temperature increases from 40°C to 50°C, thermal expansion becomes stronger, the maximum displacement increases by 15.8%, and the maximum gas pressure increases by 4.3%. Therefore, high-temperature conditions promote gas release and deformation while changing the balance between seepage-induced unloading and thermally induced stress increase.
(4) Fracture roughness directly affects permeability and gas desorption during gas migration. When the fracture roughness index decreased from 0.4 to 0.1, the maximum permeability increased by 6.18%, and the gas desorption intensity at 3000s increased by 75.7%. Smoother fractures provide more favorable flow conditions and allow stronger gas release toward the working face.

Overall, gas behavior in high-temperature coal seams with dense fracture zones cannot be interpreted only from general temperature or gas conditions. Fracture-zone distribution, fracture roughness, and thermo-mechanical deformation should be considered together when assessing gas migration and coal-rock stability near roadways and working faces. This study provides a quantitative basis for gas-hazard assessment in high-temperature mines.
