# Supplementary material for: A multi-field coupled model for gas migration in high-temperature coal seams considering fracture zones and fracture roughness
Source: PLoS One. 2026 Sep 8;21(9):e0349721. doi: 10.1371/journal.pone.0349721 (PMC13552791; doi:10.1371/journal.pone.0349721)
Supplement: Supplementary file 2 [file pone.0349721.s002.docx]

Responses to Reviewer 2

The manuscript presents an interesting coupled thermo-hydro-mechanical model for investigating gas migration and coal-rock response in high-temperature fractured coal seams. However, the discussion remains largely qualitative, and several conclusions are not sufficiently supported by quantitative analysis or field validation. The authors are encouraged to provide a clearer definition and justification of the fracture roughness parameter, include sensitivity and uncertainty analyses, and strengthen the mechanistic explanation of the observed permeability and desorption behaviors. In particular, the significant increase in gas desorption intensity compared to the relatively small permeability variation requires further clarification. Additional validation using field measurements of gas pressure, gas concentration, permeability, or deformation would substantially improve the credibility and applicability of the model predictions.

**Response:** We would like to thank you for your professional review work, constructive comments, and valuable suggestions on our manuscript. We have provided point-by-point responses to all the issue.

**1.** Thank you for this constructive comment. We have revised the numerical analysis, result descriptions, Discussion, and Conclusions to provide stronger quantitative support, including additional numerical comparisons, parameter-based analyses, and clearer interpretation of the main trends.

**2.** We have substantially revised the description of the fracture roughness parameter *χ* by adding supporting references and clarifying its physical meaning, definition basis, and connection with the equivalent permeability of rough fractures. These revisions provide a clearer justification for using *χ* to represent fracture roughness in the coupled gas-migration model.

**3.** Besides, we have added a quantitative sensitivity analysis in the revised manuscript to evaluate the effects of fracture roughness and temperature on permeability and gas desorption. The revised results show that decreasing the fracture roughness index from 0.4 to 0.1 increases the maximum permeability by 6.18%, while the gas desorption response shows a much stronger relative variation. In addition, increasing the coal-seam temperature from 294.3K to 324.3K increases the gas desorption intensity at 3000s to nearly three times its initial value and increases permeability by about 45%. These results clarify the different sensitivities of permeability and desorption to roughness and temperature. We also added a discussion of parameter uncertainty, explaining that parameter variability mainly affects the predicted magnitudes, whereas the main trends remain stable within the investigated parameter ranges.

**4.** As for the concern regarding the difference between permeability variation and gas desorption response, we have revised the related discussion to provide a clearer mechanistic explanation. In the revised manuscript, we clarify that permeability mainly represents the instantaneous flow capacity of the equivalent fracture channel and is primarily controlled by hydraulic aperture and roughness-induced flow resistance. Gas desorption intensity, however, is affected not only by permeability, but also by the matrix-fracture pressure difference, gas exchange between the coal matrix and fractures, and the cumulative desorption process. Therefore, a moderate increase in permeability can lead to a much stronger desorption response when smoother fractures maintain a larger pressure gradient between the matrix and the fracture network. We also added quantitative comparisons showing that when the fracture roughness index decreases from 0.4 to 0.1, the maximum permeability increases by 6.18%, whereas the relative difference in gas desorption intensity at 3000s reaches 75.7%. These revisions explain why gas desorption is more sensitive to fracture roughness than permeability.

**5.** We appreciate the reviewer’s constructive suggestion on strengthening the field validation of the model. In response to this suggestion, we have reorganized the original validation section and added an independent field comparison for gas-extraction seepage behavior. The original validation based on the 16035 working face is now presented as Section 4.1, while a new Section 4.2 has been added using gas-production data from the Horseshoe Canyon coalbed methane well. This additional case was used to evaluate the gas-seepage prediction capability of the proposed rough-fracture multi-field coupled model. In the revised manuscript, the simulated gas-production rate was compared with the field measurements, and quantitative error indices were added. Because the sampling times of the field data and simulation results were not identical, the simulated values were interpolated to the field measurement times, and all field points within the simulation period were included in the error calculation. The comparison gives an RMSE of 2.28×10³m³/d, an MAE of 1.70×10³m³/d, an MRE of 2.09%, and a normalized RMSE of about 2.78%. These results show that the model can reproduce the long-term decline of field gas production with acceptable accuracy. We also revised the discussion to clarify that the remaining deviations are mainly related to wellbore pressure adjustment, monitoring uncertainty, geological heterogeneity, and short-term operational disturbance. This additional validation improves the credibility of the gas-seepage component of the model and supports its application to gas migration analysis in fractured coal seams.
