# Supplementary material for: A multi-field coupled model for gas migration in high-temperature coal seams considering fracture zones and fracture roughness
Source: PLoS One. 2026 Sep 8;21(9):e0349721. doi: 10.1371/journal.pone.0349721 (PMC13552791; doi:10.1371/journal.pone.0349721)
Supplement: Supplementary file 3 [file pone.0349721.s001.docx]

**A Multi-Field Coupled Model for Gas Migration and Coal-Rock Response in High-Temperature Coal Seams with Rough Fracture Zones**

Abstract:

1. The abstract describes what was done, but it does not clearly explain what is NEW compared with existing thermo-mechanical or gas–solid coupling studies.

2. The modeling approach is too vague. Authors should mention governing numerical methods and solver used to investigate research questions.

3. The abstract states that the model was “applied” to a mining area, but it does not indicate whether the model was validated using field or experimental data.

4. Lines 14-16, The results show… stress, and displacement. The sentence should be rewritten for clarity.

5. The abstract lists many percentages but does not explain their engineering significance.

6. The conclusion is too general, make it more actionable.

1. Introduction:

1.1. Literature review is descriptive rather than critical. For example, authors should focus on what is already solved, what remains unresolved, why previous models are insufficient, how the present work advances the field.

1.2. Instead of listing papers individually, organize literature by themes. For instance, thermal management in deep mines, thermal-mechanical coupling in coal seams, fracture-controlled gas migration, roughness effects and unresolved limitations.

1.3. Redundancy: research gap is repeated multiple times (paragraphs 3 and 4). Instead of repetition, discuss why roughness matters physically, why previous simplifications may produce inaccurate predictions, and how this affects hazard assessment.

1.4. Novelty is still not sufficiently emphasized. The Introduction mentions the developed model but does not clearly distinguish it from existing coupling studies. The authors should emphasize what is fundamentally new, what specific methodological advancement is introduced.

2. Study area and engineering background:

2.1. A geographic representation of the study area could enhance the understanding of mining region.

2.2. Between Lines 105-113, authors should identify figures/numbers of the normal range of the parameters and then what are the relatively higher numbers are associated with the study area. What were the previous and latest concentrations of gas emissions? Please provide references to support the arguments.

2.3. Between Lines 114-115, any source or authentication of the reports? Authors should briefly explain the reason for change in the mine gas concentrations.

2.4. Between Lines 125-137, authors should provide geological maps indicating the locations and numbers of faults in the vicinity of study area.

2.5. Between Lines 138-147, the novelty of the study is not significant. Authors should categorically define the difference between conventional gas assessment techniques and the technique adopted by authors.

2.6. Authors have provided details about methane emissions in the mine and at working face. However, no information about mani ventilation and auxiliary ventilation has been provided to control the mine environment.

3. Multi-field coupled model for a high-temperature coal seam with rough fracture zones

3.1. What type of numerical simulation tool was used in this study? This information should be provided at the start of this section.

3.2. Line 153, “The near-roadway…” what is a high temperature coal seam? Mention the temperature considered as a high temperature in this context.

3.3. Line 158, “Unlike previous studies…” which studies are authors refereeing to, any references?

3.4. Briefly define fracture roughness index, *χ.* It is better to provide some background information for the introduced index.

4. Model validation using field observations from the 16035 fully mechanized

top-coal caving face

4.1. Between Lines 332-334, “shortest spontaneous-heating period… 23 days”, How many events were recorded? What defines “spontaneous heating”? (CO threshold? temperature? smoke?) Was 23 days measured from excavation start or from exposure of coal to airflow?

4.2. Between Lines 330-332, “maximum temperatures at 10 and 15 days are 86.5°C and 267.5°C”, a jump to 267.5°C at 15 days suggests that possible runaway reaction not explained, or numerical instability, or inconsistent boundary conditions. But the manuscript does not explain heating rate mechanism, justify why such rapid escalation occurs, or show intermediate temperature curve.

4.3. Between Lines 327-329, “oxygen is consumed more rapidly”, authors should mention oxygen concentration values (min, max, gradients), spatial oxygen maps interpretation, and comparison with field oxygen monitoring data. Without numbers, this is qualitative interpretation of a quantitative model.

4.4. The validation section should include quantitative error metrics such as relative error (%), RMSE for temperature–time profiles, and deviations in oxygen concentration between simulated and field data. It should also clearly describe the field measurement setup, including sensor types (e.g., thermocouples and gas analyzers), their spatial placement, and monitoring frequency. In addition, the study should report a sensitivity analysis to identify key parameters influencing ignition time, temperature rise rate, and oxygen depletion. These elements would improve the robustness, transparency, and credibility of the model validation.

5. Gas migration and coal-rock response in the 16035 working face

5.1. The authors should focus on discussing the results in interpretive manner rather than analytical. There should be quantitative support for the observations. For instance, in this section, gas pressure evolution (Fig. 3), temperature evolution (Fig. 4), and displacement (Fig. 5) only qualitatively described like “faster pressure transfer”, “non-uniform temperature field”, and “noticeable displacement”, respectively.

5.2. The authors state that the fracture network was generated using the methodology detailed in Section 3. However, this description lacks the necessary technical detail to evaluate the validity of the model. Specifically, the text does not clarify whether a deterministic or stochastic modeling approach was used, nor does it provide details on the fracture aperture distribution, permeability enhancement factors, or fracture connectivity validation. Without these critical parameters, the conclusions regarding "dominant gas pathways" remain highly model-dependent assumptions rather than field-validated findings. Please expand Section 3 to explicitly address these modeling choices and validation steps to substantiate the conclusions.

5.3. The temporal interpretation of the system's response is currently inconsistent and vague. For instance, the authors note that “noticeable displacement mainly appears after 5 days,” but they do not provide a clear, quantifiable threshold criterion to define what constitutes "noticeable" displacement. Furthermore, this timeline is presented in isolation, lacking a rigorous comparison with the timing of gas pressure or temperature evolution, as well as a mechanistic explanation for this apparent incubation delay. Please establish a clear baseline for the temporal thresholds used and integrate the displacement timeline with the thermal and fluid pressure data to clarify the underlying physics driving this delay.

5.4. Between Lines 430–433, the manuscript states that a larger fracture roughness index corresponds to rougher fracture surfaces and smaller effective flow space. However, the physical definition of the roughness index, its mathematical implementation in the model, and the basis for selecting values between 0.1 and 0.4 are not adequately explained. The authors should clarify whether these values are derived from field measurements, laboratory experiments, or literature sources.

5.5. Between Lines 435–436, the authors report that permeability increases by 6.18% when the fracture roughness index decreases from 0.4 to 0.1. However, only the relative change is presented. The corresponding absolute permeability values should be reported and discussed to demonstrate whether this change is significant for gas migration and mine safety applications.

5.6. Between Lines 443–449, the manuscript reports a 75.7% increase in gas desorption intensity when the fracture roughness index decreases from 0.4 to 0.1. This increase appears disproportionately large compared with the reported 6.18% increase in permeability (Lines 435–436). The authors should provide a more detailed explanation of the coupled mechanisms responsible for this behavior and quantify the relative contributions of permeability enhancement and pressure-gradient evolution.

5.7. Between Lines 424–472, the analysis focuses exclusively on fracture roughness and temperature effects, but no sensitivity analysis is provided. Given the large number of model parameters listed in Table 2, the authors should identify which parameters most strongly influence permeability, desorption intensity, gas pressure, temperature evolution, and coal-rock deformation. This would improve confidence in the robustness of the model predictions.

6. Conclusion

6.1. Between Lines 479-485, the statement that the field comparison "supports the reasonableness of the coupled treatment and parameter setting" may be overstated because validation is based primarily on spontaneous-heating time and the extent of the high-temperature zone. Additional validation metrics, such as temperature evolution, oxygen concentration, gas pressure, and deformation measurements, would be needed to fully support this conclusion.

6.2. Between Lines 486-492, the manuscript concludes that fracture networks control the spatial distributions of gas pressure, temperature, stress, and displacement. However, this conclusion is largely based on qualitative observations from simulation figures. Quantitative indicators (e.g., pressure gradients, flow velocities, stress concentrations, or displacement magnitudes) should be reported to support these statements.

6.3. Between Lines 489-491, the statement that "part of the stress carried by the solid matrix is transferred to the gas in the fracture zones" is physically unclear. The authors should reformulate this explanation using the framework of effective stress and pore pressure evolution, which would provide a more rigorous interpretation of the observed stress reduction.

6.4. Between Lines 493-499, the conclusions regarding the influence of temperature on gas migration and coal-rock stability are based on a relatively narrow temperature range (40–50°C). The authors should discuss whether these findings remain valid under broader temperature conditions commonly encountered in high-temperature mines.
