# Supplementary material for: A multi-field coupled model for gas migration in high-temperature coal seams considering fracture zones and fracture roughness
Source: PLoS One. 2026 Sep 8;21(9):e0349721. doi: 10.1371/journal.pone.0349721 (PMC13552791; doi:10.1371/journal.pone.0349721)
Supplement: Peer review history

# Peer review: Decision Letter 0

**DOI:** 10.1371/journal.pone.0349721.r001

**Authors:** Baohua Guo

10 Jun 2026

Dear Dr. Jiang,

Thank you for submitting your manuscript to PLOS ONE. After careful consideration, we feel that it has merit but does not fully meet PLOS ONE’s publication criteria as it currently stands. Therefore, we invite you to submit a revised version of the manuscript that addresses the points raised during the review process.

Please submit your revised manuscript by Aug 09 2026 11:59PM. If you will need more time than this to complete your revisions, please reply to this message or contact the journal office at plosone@plos.org. When you're ready to submit your revision, log on to https://www.editorialmanager.com/pone/ and select the 'Submissions Needing Revision' folder to locate your manuscript file.

- A letter that responds to each point raised by the academic editor and reviewer(s). You should upload this letter as a separate file labeled 'Response to Reviewers'.
- A marked-up copy of your manuscript that highlights changes made to the original version. You should upload this as a separate file labeled 'Revised Manuscript with Track Changes'.
- An unmarked version of your revised paper without tracked changes. You should upload this as a separate file labeled 'Manuscript'.

If you would like to make changes to your financial disclosure, please include your updated statement in your cover letter. Guidelines for resubmitting your figure files are available below the reviewer comments at the end of this letter.

If applicable, we recommend that you deposit your laboratory protocols in protocols.io to enhance the reproducibility of your results. Protocols.io assigns your protocol its own identifier (DOI) so that it can be cited independently in the future. For instructions see: https://journals.plos.org/plosone/s/submission-guidelines#loc-laboratory-protocols. Additionally, PLOS ONE offers an option for publishing peer-reviewed Lab Protocol articles, which describe protocols hosted on protocols.io. Read more information on sharing protocols at https://plos.org/protocols?utm_medium=editorial-email&utm_source=authorletters&utm_campaign=protocols.

As the corresponding author, your ORCID iD is verified in the submission system and will appear in the published article. PLOS supports the use of ORCID, and we encourage all coauthors to register for an ORCID iD and use it as well. Please encourage your coauthors to verify their ORCID iD within the submission system before final acceptance, as unverified ORCID iDs will not appear in the published article. *Only*  the individual author can complete the verification step; PLOS staff *cannot*  verify ORCID iDs on behalf of authors.

We look forward to receiving your revised manuscript.

Kind regards,

Baohua Guo

Academic Editor

PLOS One

Journal Requirements:

When submitting your revision, we need you to address these additional requirements.

1. Please ensure that your manuscript meets PLOS ONE's style requirements, including those for file naming. The PLOS ONE style templates can be found at

https://journals.plos.org/plosone/s/file?id=wjVg/PLOSOne_formatting_sample_main_body.pdf and

https://journals.plos.org/plosone/s/file?id=ba62/PLOSOne_formatting_sample_title_authors_affiliations.pdf

2. Please note that PLOS One has specific guidelines on code sharing for submissions in which author-generated code underpins the findings in the manuscript. In these cases, we expect all author-generated code to be made available without restrictions upon publication of the work. Please review our guidelines at https://journals.plos.org/plosone/s/materials-and-software-sharing#loc-sharing-code and ensure that your code is shared in a way that follows best practice and facilitates reproducibility and reuse.

3. In this instance it seems there may be acceptable restrictions in place that prevent the public sharing of your minimal data. However, in line with our goal of ensuring long-term data availability to all interested researchers, PLOS’ Data Policy states that authors cannot be the sole named individuals responsible for ensuring data access (http://journals.plos.org/plosone/s/data-availability#loc-acceptable-data-sharing-methods).

Data requests to a non-author institutional point of contact, such as a data access or ethics committee, helps guarantee long term stability and availability of data. Providing interested researchers with a durable point of contact ensures data will be accessible even if an author changes email addresses, institutions, or becomes unavailable to answer requests.

Before we proceed with your manuscript, please also provide non-author contact information (phone/email/hyperlink) for a data access committee, ethics committee, or other institutional body to which data requests may be sent. If no institutional body is available to respond to requests for your minimal data, please consider if there any institutional representatives who did not collaborate in the study, and are not listed as authors on the manuscript, who would be able to hold the data and respond to external requests for data access? If so, please provide their contact information (i.e., email address). Please also provide details on how you will ensure persistent or long-term data storage and availability

4. Please include your full ethics statement in the ‘Methods’ section of your manuscript file. In your statement, please include the full name of the IRB or ethics committee who approved or waived your study, as well as whether or not you obtained informed written or verbal consent. If consent was waived for your study, please include this information in your statement as well.

5. If the reviewer comments include a recommendation to cite specific previously published works, please review and evaluate these publications to determine whether they are relevant and should be cited. There is no requirement to cite these works unless the editor has indicated otherwise.

[Note: HTML markup is below. Please do not edit.]

Reviewers' comments:

Reviewer's Responses to Questions

**Comments to the Author**

1. Is the manuscript technically sound, and do the data support the conclusions?

Reviewer #1: Partly

Reviewer #2: Partly

**********

2. Has the statistical analysis been performed appropriately and rigorously?

Reviewer #1: N/A

Reviewer #2: Yes

**********

3. Have the authors made all data underlying the findings in their manuscript fully available?

The PLOS Data policy

Reviewer #1: No

Reviewer #2: Yes

**********

4. Is the manuscript presented in an intelligible fashion and written in standard English?

Reviewer #1: No

Reviewer #2: Yes

**********

Reviewer #1: This manuscript addressed a practically important problem: gas migration and coal-rock stability in high-temperature coal mines with dense fracture zones. The authors developed a multi-physical coupled model which incorporates gas seepage, heat transfer, coal-rock deformation, and gas desorption, and introduce a fracture roughness index to characterize the effect of fracture surface morphology on permeability. The model was applied to the 16035 working face in the Liupanshui mining area, Guizhou, China, and yielded several quantitative results of engineering relevance. The writing-up of this manuscript is generally understandable, but some clarifications and improvements are necessary. My particular comments are as follows.

1, The novelty should be clearly described. The model is an assemblage of previous model. The only difference is to introduce the fracture roughness index, but the physical meanings and introduction of this index are not based on solid physical or mathematical development.

2, In Equations (5)–(8), the fracture roughness index χ = √2·γ/a_i (γ = fracture height, a_i = initial fracture aperture). However, the physical meaning and typical range of γ and a_i are not explained; The relationship between χ and established roughness characterizations (JRC, fractal dimension, self-affine fractals) is not discussed.

3, Several derivations are difficult to follow: The structure of porosity equation (11) is not intuitive; the combination of variables in the exponent is not explained; The physical meaning of terms in Equation (14) is unclear (e.g., H·k·∂ε_v/∂t); Parameters in Equation (21) (e.g., q_ad) are not defined.

4, The model was validated using coal spontaneous combustion (temperature, oxygen concentration), not the core gas migration behavior. This is a logical inconsistency - using a secondary phenomenon to validate a model intended for a primary phenomenon is not convincing.

5, Section 5.1 states "rapid flow in the early stage causes a short-term stress decrease." Fig. 6(a) does show a decrease, but the magnitude (1–2 MPa) is small relative to the total stress (8–12 MPa). Is this conclusion robust?

6, There are many sentences >35 words, especially in Introduction and Methods. There is also non-standard English. [5] is in the reference list but not cited in the context. “fracture zone” / “fracture zones” / “rough fracture zones” used interchangeably. Lines 72–77 and 78–83 in Introduction are highly repetitive.

7, Some Figures are required for improvement. Figures 2–9 have minimal titles (e.g., “Fig. 5 Temporal and spatial evolution of displacement…”). Missing explanations of colors, symbols, and units.

8, Reference list has formatting problems such as et al. in author list, [J], [C], [M]. Please follow the Journal format.

9, English and presentations should be significantly improved for clear and correct statements or expressions.

Reviewer #2: The manuscript presents an interesting coupled thermo-hydro-mechanical model for investigating gas migration and coal-rock response in high-temperature fractured coal seams. However, the discussion remains largely qualitative, and several conclusions are not sufficiently supported by quantitative analysis or field validation. The authors are encouraged to provide a clearer definition and justification of the fracture roughness parameter, include sensitivity and uncertainty analyses, and strengthen the mechanistic explanation of the observed permeability and desorption behaviors. In particular, the significant increase in gas desorption intensity compared to the relatively small permeability variation requires further clarification. Additional validation using field measurements of gas pressure, gas concentration, permeability, or deformation would substantially improve the credibility and applicability of the model predictions.

**********

what does this mean?). If published, this will include your full peer review and any attached files.

If you choose “no”, your identity will remain anonymous but your review may still be made public.

**Do you want your identity to be public for this peer review?**  For information about this choice, including consent withdrawal, please see our Privacy Policy

Reviewer #1: No

Reviewer #2: No

**********

[NOTE: If reviewer comments were submitted as an attachment file, they will be attached to this email and accessible via the submission site. Please log into your account, locate the manuscript record, and check for the action link "View Attachments". If this link does not appear, there are no attachment files.]

To ensure your figures meet our technical requirements, please review our figure guidelines: https://journals.plos.org/plosone/s/figures

You may also use PLOS’s free figure tool, NAAS, to help you prepare publication quality figures: https://journals.plos.org/plosone/s/figures#loc-tools-for-figure-preparation.

NAAS will assess whether your figures meet our technical requirements by comparing each figure against our figure specifications.

Attachment Submitted filename: PONE-D-26-21712_ARQ (10-06-2026).docx

# Author comment: Author response to Decision Letter 1

**DOI:** 10.1371/journal.pone.0349721.r002

4 Jul 2026

We have provided point-by-point responses to all reviewer and editor comments. The detailed response letter and the revised manuscript have been uploaded as attachments.

Attachment Submitted filename: Responses to Reviewer 2.docx

# Peer review: Decision Letter 1

**DOI:** 10.1371/journal.pone.0349721.r003

**Authors:** Baohua Guo

15 Jul 2026

A Multi-Field Coupled Model for Gas Migration in High-Temperature Coal Seams Considering Fracture Zones and Fracture Roughness

PONE-D-26-21712R1

Dear Dr. Jiang,

We’re pleased to inform you that your manuscript has been judged scientifically suitable for publication and will be formally accepted for publication once it meets all outstanding technical requirements.

Within one week, you’ll receive an e-mail detailing the required amendments. When these have been addressed, you’ll receive a formal acceptance letter and your manuscript will be scheduled for publication.

An invoice will be generated when your article is formally accepted. Please note, if your institution has a publishing partnership with PLOS and your article meets the relevant criteria, all or part of your publication costs will be covered. Please make sure your user information is up-to-date by logging into Editorial Manager at Editorial Manager® and clicking the ‘Update My Information' link at the top of the page. For questions related to billing, please contact billing support.

If your institution or institutions have a press office, please notify them about your upcoming paper to help maximize its impact. If they’ll be preparing press materials, please inform our press team as soon as possible -- no later than 48 hours after receiving the formal acceptance. Your manuscript will remain under strict press embargo until 2 pm Eastern Time on the date of publication. For more information, please contact onepress@plos.org.

Kind regards,

Baohua Guo

Academic Editor

PLOS One

Additional Editor Comments (optional):

Reviewers' comments:

Reviewer's Responses to Questions

**Comments to the Author**

Reviewer #1: All comments have been addressed

**********

2. Is the manuscript technically sound, and do the data support the conclusions?

Reviewer #1: Yes

**********

3. Has the statistical analysis been performed appropriately and rigorously?

Reviewer #1: N/A

**********

4. Have the authors made all data underlying the findings in their manuscript fully available?

The PLOS Data policy

Reviewer #1: Yes

**********

5. Is the manuscript presented in an intelligible fashion and written in standard English?

Reviewer #1: Yes

**********

Reviewer #1: There are some writing-ups for improvements: In Table 2: phi_0 has no unit (current m^{2}. In Table 3, permeability unit: m^{2} or mD. Please use the same unit in your paper.

**********

what does this mean?). If published, this will include your full peer review and any attached files.

If you choose “no”, your identity will remain anonymous but your review may still be made public.

**Do you want your identity to be public for this peer review?**  For information about this choice, including consent withdrawal, please see our Privacy Policy

Reviewer #1: No

**********

# Editor report: Acceptance letter

**DOI:** 10.1371/journal.pone.0349721.r004

**Authors:** Baohua Guo

PONE-D-26-21712R1

PLOS One

Dear Dr. Jiang,

I'm pleased to inform you that your manuscript has been deemed suitable for publication in PLOS One. Congratulations! Your manuscript is now being handed over to our production team.

At this stage, our production department will prepare your paper for publication. This includes ensuring the following:

* All references, tables, and figures are properly cited

* All relevant supporting information is included in the manuscript submission,

* There are no issues that prevent the paper from being properly typeset

You will receive further instructions from the production team, including instructions on how to review your proof when it is ready. Please keep in mind that we are working through a large volume of accepted articles, so please give us a few days to review your paper and let you know the next and final steps.

Lastly, if your institution or institutions have a press office, please let them know about your upcoming paper now to help maximize its impact. If they'll be preparing press materials, please inform our press team within the next 48 hours. Your manuscript will remain under strict press embargo until 2 pm Eastern Time on the date of publication. For more information, please contact onepress@plos.org.

You will receive an invoice from PLOS for your publication fee after your manuscript has reached the completed accept phase. If you receive an email requesting payment before acceptance or for any other service, this may be a phishing scheme. Learn how to identify phishing emails and protect your accounts at https://explore.plos.org/phishing.

If we can help with anything else, please email us at customercare@plos.org.

Thank you for submitting your work to PLOS ONE and supporting open access.

Kind regards,

PLOS ONE Editorial Office Staff

on behalf of

Dr. Baohua Guo

Academic Editor

PLOS One
